# Effectiveness and safety of motion-style acupuncture treatment using traction for inpatients with acute low back pain caused by a traffic accident: A randomized controlled trial

**DOI:** 10.1097/MD.0000000000038590

**Published:** 2024-06-21

**Authors:** Byung-Hak Park, Jeong-Hun Han, Jin-Hun Park, Tae-Woon Min, Hyun-Jun Lee, Yoon Jae Lee, Sook-Hyun Lee, Kyoung Sun Park, In-Hyuk Ha

**Affiliations:** a Jaseng Hospital of Korean Medicine, Seoul, Republic of Korea; b Jaseng Spine and Joint Research Institute, Jaseng Medical Foundation, Seoul, Republic of Korea.

**Keywords:** acupuncture, low back pain, motion-style acupuncture treatment, traction, traffic accident

## Abstract

**Background:**

Musculoskeletal symptoms, such as neck pain and low back pain (LBP) are common after a traffic accident (TA). While motion-style acupuncture treatment (MSAT) is effective in relieving pain, MSAT using traction (T-MSAT) has rarely been studied, and evidence for its efficacy and safety is lacking. To address this gap, this study aimed to assess the effectiveness and safety of T-MSAT for pain and functional disturbances in patients with acute LBP caused by a TA.

**Methods:**

This two-armed, parallel, assessor blinded randomized controlled trial, conducted at Jaseng Hospital of Korean Medicine, included 100 patients with acute LBP occurring within 1 week of a TA. The participants were randomly allocated (1:1 ratio) to receive either combined T-MSAT and integrative Korean medicine treatment (IKMT) or only conventional IKMT, applied for 3 consecutive days after admission. The primary outcome was the difference between numerical rating scale (NRS) scores for LBP at baseline and after treatment completion on day 4 after admission.

**Results:**

At the primary endpoint, the difference in NRS scores for LBP between the T-MSAT and control groups was 0.94 (95% confidence interval [CI] 0.40–1.48). The T-MSAT group showed a significantly lower NRS score for LBP than the control group. Differences in visual analogue scale (VAS) scores between the T-MSAT and control groups were significant at baseline and discharge. The area under the curve of the VAS score showed a significant difference (−46.86 [95% CI −85.13 to −8.59]), indicating faster pain reduction in the T-MSAT group than in the control group. Recovery (30% pain reduction) was achieved more rapidly in the T-MSAT group than in the control group (log-rank test *P* = .005). Meanwhile, the NRS, VAS, Oswestry disability index, and quality of life scores at discharge or at the 12-week follow-up showed no significant difference. The rates of mild adverse events (AEs) were comparable between the groups. No severe AEs were reported, and none of the AEs were associated with the clinical trial.

**Conclusions:**

T-MSAT combined with IKMT is a safe treatment that can effectively and quickly reduce initial pain in patients with LBP.

## 1. Introduction

The number of personal automobiles in Korea has increased exponentially, with a consequent increase in the number of traffic accidents (TAs) and people injured.^[1]^ TAs may have diverse consequences, including musculoskeletal injuries^[2,3]^; neck pain and low back pain (LBP) commonly develop after a TA.^[4]^ Approximately 37% of patients experience severe LBP even 6 weeks after the TA.^[2]^ Additionally, 60.4% of patients who received treatment within 30 days of a TA reported LBP.^[5]^ Acute LBP caused by a TA could easily become chronic, as evidenced by one study reporting that 31% of patients experience LBP up to 1 year after the initial injury.^[6]^ Thus, acute LBP caused by a TA is more likely to progress to chronic LBP than regular acute LBP.^[6]^

LBP is one of the most common disorders worldwide, experienced by 50% to 80% of individuals at least once during their lifetime.^[7]^ Due to its high recurrence rate, LBP endlessly affects the quality of life of an individual.^[8,9]^ According to the Global Burden of Disease study (1990–2017), the global population experiencing LBP has increased significantly over the past 3 decades. Consequently, the years lost to disability have also increased significantly.^[10]^ Persistent LBP can cause activity limitation and occupational disruption, resulting in social and economic losses.^[11,12]^

In more than half of the cases, pain recurs within 1 year of acute LBP onset, and the recurrence rate can reach 75%.^[13]^ Furthermore, up to 33% and 15% of these patients continue to experience moderate and severe pain, respectively.^[13]^ Therefore, timely treatment for acute LBP is crucial to prevent its progression to chronic LBP. Recommended treatments for acute LBP include drugs (analgesics and herbal medicine), exercise, acupuncture, electromyographic biofeedback, epidural steroid injection, and spinal manipulation.^[13,14]^ Guidelines for acute LBP recommend that patients continue performing their routine activities, such as walking, and avoid bed rest.^[14]^ Several studies indicate the effectiveness of traction therapy in the treatment of LBP.^[15]^

Motion-style acupuncture treatment (MSAT) is a therapeutic modality in which passive or active movements are performed with acupuncture needles in the body.^[16]^ In Korea and China, MSAT has shown greater effectiveness than conventional acupuncture therapy in reducing pain and improving function in patients with musculoskeletal disorders.^[16–20]^ MSAT using traction (T-MSAT) is a type of MSAT used to treat musculoskeletal issues in the lumbar region, such as lumbar sprain and lumbar disc herniation. During T-MSAT, the patient walks with the inserted acupuncture needles after traction is applied to the body using a device. Consequently, MSAT, which incorporates walking and acupuncture therapy, may be a feasible treatment option for patients with acute LBP. MSAT has been reported to be at least 5 times more effective at relieving pain than analgesic injection.^[16]^ However, T-MSAT remains underexplored, and evidence for its efficacy and safety is lacking. Consequently, the purpose of this randomized controlled trial (RCT) was to evaluate the effectiveness and safety of T-MSAT in patients with acute LBP resulting from TAs.

## 2. Methods

This study was conducted in accordance with the Consolidated Standards of Reporting Trials (CONSORT) 2010 and Standards for Reporting Interventions in Clinical Trials of Acupuncture (STRICTA) reporting guidelines. The study investigated the LBP score, lumbar dysfunction index, quality of life, posttraumatic stress after an accident, and adverse events (AEs). The objective was to assess the effectiveness and safety of combined treatment with T-MSAT and conventional inpatient integrative Korean medicine treatment (IKMT) compared with those of IKMT alone. The trial protocol was approved by the Institutional Review Board of Jaseng Hospital of Korean Medicine (IRB 2020-08-011) prior to participant recruitment. Each participant provided written informed consent after receiving information regarding the trial during the first visit. The trial was registered at clinicaltrials.gov (NCT04554446), and the progress of the trial was continuously updated.

### 2.1. Study design

This was a two-armed, parallel, single-blinded (outcome assessor blinded) RCT. The clinical trial was conducted at Jaseng Hospital of Korean Medicine. One hundred patients with acute LBP occurring within 1 week of a TA were recruited.

Inpatients hospitalized at Jaseng Hospital of Korean Medicine for injuries caused by a TA were recruited through posters placed inside the hospital and on the hospital’s website, as well as via recommendations from physicians. The first and last patients were enrolled on September 21, 2020 and March 15, 2021, respectively. Those who voluntarily agreed to participate in the study completed and submitted an informed consent form, which explained the nature of the T-MSAT treatment. It specified that participants in this study would be randomly assigned to either the IKMT group, which involved routine treatment performed during hospitalization, or the group receiving additional T-MSAT in conjunction with IKMT. Subsequently, the screening researcher enrolled 100 patients. The inclusion criteria comprised age between 19 and 70 years, numerical rating scale (NRS) score for LBP ≥ 5, requirement of inpatient treatment for acute LBP caused by a TA that occurred within 1 week of the TA, and voluntary agreement to participate in the study and submission of an informed consent form. The exclusion criteria were the presence of any condition other than TA that can cause acute LBP, such as fracture and spinal metastasis; progressive neurologic deficits; soft tissue disorders, such as fibromyalgia and gout; chronic diseases; pregnancy; surgery of the lumbar spine within 3 weeks prior to enrollment; and severe mental disorders.

Participants were required to attend a total of 6 visits, including the first day of hospitalization. The surveys were conducted through face-to-face interviews, except the 12-week follow-up survey, which was replaced with a telephone interview. On the second day of hospitalization, participants (N = 100) were randomly allocated (1:1 ratio) to the group that received combined treatment with T-MSAT and IKMT (T-MSAT group, n = 50) or the group that received only conventional IKMT (control group, n = 50). The management of the participants and the treatment processes followed those detailed in the trial protocol.

### 2.2. Randomisation and masking

A statistician not directly involved in the study used SAS version 9.4 (SAS Institute, Inc., Cary) for block randomization to assign an equal number of participants to each group (50 per group), with random block sizes of 2, 4, or 6. Randomisation was applied to all 100 patients according to the single-center patient recruitment and enrollment protocol. The generated randomization results were sealed in envelopes and kept in a locked locker. An envelope was opened in random order in the presence of each participant before the treatment for the 1:1 group allocation. Subsequently, the random allocation number was recorded on the electronic chart. Once the allocation was made, no further change was possible.

### 2.3. Blinding

Because the study design did not allow blinding of both the patients and therapists to group allocation, a single-blinded design (assessor blinded) was applied. An assessor did not participate in the intervention, and a researcher who was blinded to the group allocation performed the assessment in a separate area prior to the intervention.

### 2.4. Interventions

#### 2.4.1. Control group

The control group only received conventional IKMT, consisting of acupuncture, Chuna therapy, pharmacopuncture, and herbal medicine (decoction). Detailed information on acupuncture is presented in Supplemental Digital Content (see Table S1, Supplemental Digital Content, http://links.lww.com/MD/M952, which provides information on acupuncture), in accordance with STRICTA standards.

#### 2.4.2. Experimental group

The experimental (T-MSAT) group received both conventional IKMT and T-MSAT. The intervention was administered by a physician trained in the T-MSAT protocol. The participants in the T-MSAT group underwent 3 sessions of T-MSAT, once daily between days 2 and 4. They also received the same IKMT as the control group. A doctor of Korean medicine, possessing over 18 months of clinical experience, utilized a traction device on the patient’s body. Following this, acupuncture needles were inserted into 7 acupoints, including LI11 (bilateral), LR2 (bilateral), EX-B2 (bilateral), and GV16 (unilateral), with de qi sensation, using 0.30 × 0.40 mm disposable acupuncture needles (Dongbang Medical, Boryeong, Korea). Details of acupuncture are presented in Supplemental Digital Content (see Table S1, Supplemental Digital Content, http://links.lww.com/MD/M952, which provides information on acupuncture), in accordance with STRICTA standards. Subsequently, the doctor instructed the patient to walk for approximately 15 minutes. The walking speed, needle stimulation strength, and traction were adjusted depending on the pain or discomfort experienced by the patient.

### 2.5. Co-interventions

Participants were not restricted from utilizing other treatments that may have influenced the assessment of LBP when deemed necessary owing to severe pain experienced during the study (including the treatment and follow-up periods). However, the participants were required to immediately notify the investigator of any co-interventions used, which were then checked and recorded.

### 2.6. Outcome measures

The outcomes were measured at each time point during the 12-week study period. The primary endpoint was assessed after treatment completion on day 4 (day 4-2).

### 2.7. Primary outcome

The primary outcome was the difference between the NRS scores for LBP measured at admission and at the end of the treatment on day 4-2. The NRS is used to express subjective pain felt by the patient as an objective numerical value. Patients were instructed to select a number between 0 (no pain) and 10 (worst pain imaginable) that best described their current pain level.^[21,22]^

### 2.8. Secondary outcomes

Secondary outcomes were assessed using the visual analogue scale (VAS) scores for LBP and radiating leg pain, NRS score for radiating leg pain, lumbar active range of motion (aROM), and Oswestry disability index (ODI), 12-item short-form survey (SF-12), posttraumatic stress disorder checklist for DSM-5 (PCL-5-K), and patient global impression of change (PGIC) scores. Moreover, a follow-up investigation was conducted 12 weeks after enrollment in the study. It involved assessing the NRS scores for LBP and radiating leg pain, ODI, SF-12, PCL-5-K, and PGIC. Furthermore, additional radiological findings from lumbar imaging after the TA (radiography, magnetic resonance imaging, and computed tomography) were recorded, and accident settlement, treatment completion, and return to work status were investigated. The schedule for outcome assessment is presented in Table 1.

**Table 1:** Schedule of the study.

Time point	Study period					
	Screening	Active treatment				F/U
	Day 1 of hospitalization	Day 2 (visit 1)	Day 3 (visit 2)	Day 4 (visit 3)	Discharge (visit 4)	12 weeks after registration (visit 5)
Enrolment						
Eligibility screening	○					
Written informed consent	○					
Sociodemographic characteristics	○					
TA-related information*	○					
Medical history (post-LBP and other)	○					
Physical examination	○					
Randomised allocation		○				
Credibility and expectancy questionnaire		○				
Intervention						
T-MSAT + IKMT	△ (only IKMT)	○	○	○	△ (only IKMT)	
IKMT	○	○	○	○	○	
Assessment						
Symptom evaluation and medication change		○	○	○	○	○
NRS of LBP	○	○§	○	○§	○	○
VAS of LBP		○‖	○	○‖	○	
ROM of the lumbar spine		○¶	○	○¶	○	
NRS of leg pain		○#	○	○#	○	○
VAS of leg pain		○**	○	○**	○	
ODI		○		○††	○	○
SF-12		○		○††	○	○
PGIC				○††	○	○
PCL-5-K		○		○††	○	○
Diagnostic imaging†						○
Details of F/U‡						○
Adverse events		○	○	○	○	○

If the dates of visits 3 and 4 coincided, the assessment for day 4 could be replaced by discharge assessment. The follow-up visit at 12 weeks after registration had a time window of ±14 days.

F/U = follow-up, IKMT = integrative Korean medicine therapy, LBP = low back pain, NRS = numerical rating scale, ODI = Oswestry disability index, PCL-5-K = posttraumatic stress disorder checklist for DSM-5, PGIC = patient global impression of change, ROM = range of motion, SF-12 = 12-item short-form health survey, TA = traffic accident, T-MSAT = motion-style acupuncture therapy using traction, VAS = visual analogue scale.

* Date of accident, severity of accident, primary diagnosis, etc.

† Confirmation of imaging, including lumbar-spine radiography, computed tomography, and magnetic resonance imaging.

‡ Settlement status, completion of treatment, and return to work status.

§ Additional NRS of low back pain after treatment.

‖ Additional VAS of low back pain after treatment.

¶ Additional ROM of the lumbar spine after treatment.

# Additional NRS of leg pain after treatment.

** Additional VAS of leg pain after treatment.

†† Investigated only if the dates of visits 3 (day 4) and 4 (discharge) coincided.

### 2.9. AEs

AEs were investigated by the researcher during each visit and documented on the electronic medical record by both the physician and researcher, both before and after the treatment, without exception. The researcher assessed the causality between each treatment method and all AEs based on a six-step scale according to the World Health Organization-Uppsala Monitoring Centre causality assessment system.^[23]^ AE severity was classified into 3 levels according to the Spilker classification^[24]^: mild, when no treatment was required and the AE did not significantly affect the normal life (function) of the participant; moderate, an AE that significantly affected the normal life (function) of the participant and may have required treatment, with potential recovery after the treatment; and severe, a serious AE necessitating advanced treatment and potentially resulting in sequelae. The frequency and severity of AEs were examined and compared between the groups to assess the safety of the treatments.

### 2.10. Sample size

The null hypothesis of the study posited no difference in pain between the experimental and control groups. To evaluate this hypothesis, analysis of covariance was utilized as the primary analysis.

The parameters were set to a significance level of α = 0.05 (two-sided test), type 2 error (β) of 0.2, and a statistical power of 80%. A previous study used an effect size of 0.58 to determine the sample size.^[16]^ Considering that we planned to use analysis of covariance as the primary analysis and assuming a correlation of 0.5, the minimum required sample size for each group was 37.^[25]^ As patients from TAs can be discharged early, the objective was to enroll 100 participants in total for both groups, assuming a 25% dropout rate.

### 2.11. Patient and public involvement

Neither patients nor the public were involved in the development of the research questions, selection of the outcome measures, design of the study, or study conduct.

### 2.12. Data management and analysis

#### 2.12.1. Data collection and management

All records were maintained on paper and stored in case files. Additionally, a standard internal operating procedure was prepared to train all researchers, including assessors and physicians, on care report writing, data input, and interventional procedures. Data recorded in the case reports were entered by an internal researcher using the double data entry method. After double checking, access to the data was blocked to all researchers except the data manager. The individuals’ personal information was not acquired during the study, and participant information was recorded via de-identification coding. Data collection commenced on September 21, 2020, at the time of the first patient enrollment, and concluded on June 15, 2021, the final follow-up date for the last enrolled participant. Data analysis for the study commenced on July 7, 2021, after the completion of data double entry and monitoring.

#### 2.12.2. Statistical analysis

Both intention-to-treat (ITT) and per-protocol (PP) analyses were conducted in this study. The ITT analysis was the primary analysis, with the PP analysis being a separate analysis of participants who received at least 3 treatment sessions. Missing values were primarily processed by multiple imputation, and the statistician was blinded. Additionally, sensitivity analyses were conducted using the mixed model repeated measures and last observation carried forward (LOCF) methods.

The demographic characteristics of the participants and their treatment expectancy were assessed for each group. Continuous variables are expressed as mean (standard deviation) or median (quartile), and differences between the 2 groups were compared using Student *t* test. Categorical variables are expressed as frequency (%) and were evaluated using the chi-square test or Fisher’s exact test.

The efficacy endpoints were the amount of change in continuous outcomes (NRS, VAS, ODI, SF-12, aROM, and PCL-5-K scores) at various time points relative to the baseline. Analysis of covariance was the primary analysis, with the pretreatment (baseline) values of each variable and age as covariates and the group as a fixed factor. For additional analysis, a linear mixed model was employed. This model included fixed effects for group and visit, a random effect for individual patients, and covariates of age and baseline measurements. The model utilized an unstructured covariance structure to calculate the change in outcomes from baseline. To compare the cumulative total of each outcome value over the study period (treatment period and entire study period) for the 2 groups, the areas under the curve (AUCs) for each time point after random allocation were calculated and compared using the trapezoidal rule. Furthermore, the percentage of patients at time points where the VAS score for LBP decreased by ≥ 30% relative to the baseline was compared and analyzed. The Cox proportional hazard model was utilized to compare hazard ratios. Furthermore, exploratory subgroup analyses were conducted to compare the differences in the level of pain improvement between the T-MSAT and control groups. All statistical analyses were performed using SAS 9.4 (SAS Institute, Inc.) or R studio 1.1.463 (2009–2018 RStudio, Inc.), with significance set at *P* < .05.

## 3. Results

**Figure 1.**
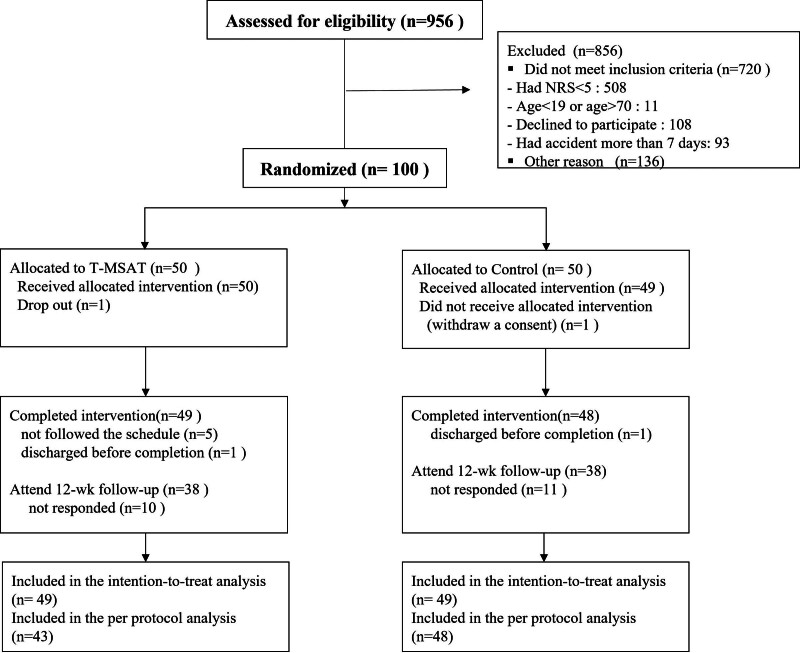
Consolidated Standards of Reporting Trials flow chart of patients. NRS = numerical rating scale; T-MSAT = motion-style acupuncture treatment using traction.

Figure 1 depicts participant recruitment and follow-up. Between September 21, 2020 and March 15, 2021, 956 patients who complained of LBP following a TA were considered for enrollment. Among these, 856 patients were excluded: 720 patients did not meet the inclusion criteria and 136 met the exclusion criteria (Fig. 1).

The study population comprised 100 participants, who were then randomly allocated into the MSAT (n = 50) and control (n = 50) groups. Subsequently, one participant in the control group withdrew consent before the first intervention and was therefore dismissed. In the T-MSAT group, one participant was excluded because they met the stopping rule outlined in the protocol. Consequently, the ITT analysis set included 49 participants in each group.

At the 12-week follow-up, 38 participants from the T-MSAT group, excluding 10 patients who did not respond to the follow-up request and 1 dropout, and 38 participants from the control group, excluding 10 patients who did not respond to the follow-up request, participated in the follow-up (Fig. 1). For the PP analysis, 43 participants in the T-MSAT group, excluding 6 patients who did not complete the 3 T-MSAT sessions, and 48 participants in the control group, excluding 1 patient who did not complete the 3 IKMT sessions, were included.

### 3.1. Baseline characteristics

Table 2 presents the baseline characteristics of the participants. The percentages of women in the T-MSAT and control groups were 46.9% and 55.1%, respectively. The mean ages in the T-MSAT and control groups were 35.2 ± 10.1 and 39.9 ± 13.2 years, respectively, indicating that the patients in the T-MSAT group were slightly younger (*P* = .049). Apart from age, no other significant differences in baseline characteristics were observed between the 2 groups (Table 2). The credibility and expectancy scores in the T-MSAT (6.2 ± 1.5) and control (7.4 ± 1.2) groups were significantly different (*P* < .001).

**Table 2:** Baseline characteristics of participants.

	T-MSAT (n = 49)	Control (n = 49)	*P* value
Sex, n (%)			
Female	23 (46.9)	27 (55.1)	.54
Male	26 (53.1)	22 (44.9)	
Age (years)	32.0 [28.0–40.0]	36.0 [29.0; 52.0]	.12
Height (cm)	170.0 (9.1)	167.2 (9.5)	.15
Weight (kg)	69.0 [57.0; 80.0]	67.0 [55.0; 78.0]	.66
BMI (kg/m^2^)	23.9 [20.5; 26.3]	23.4 [20.8; 26.5]	.89
WAD			
WAD I	28 (57.1)	23 (46.9)	.42
WAD II	21 (42.9)	26 (53.1)	
Onset (days)	3.0 [2.0; 5.0]	3.0 [2.0; 4.0]	.44
History of LBP	19 (38.8)	17 (34.7)	.83
Positive results of PE	36 (73.5)	35 (71.4)	1
NRS (LBP)	6.0 [5.0; 7.0]	6.0 [5.0; 7.0]	.82
NRS (RP)	5.0 [3.0; 6.0]	5.0 [3.0; 6.0]	.55
VAS (LBP)	61.3 (10.2)	60.4 (10.7)	.68
VAS (RP)	50.0 [20.0; 61.0]	50.0 [33.0; 63.0]	.56
ROM (FLX)	70.0 [45.0; 90.0]	60.0 [45.0; 90.0]	.80
ROM (EXT)	20.0 [10.0; 20.0]	10.0 [10.0; 20.0]	.45
ROM (RLF)	30.0 [20.0; 30.0]	30.0 [20.0; 30.0]	.51
ROM (LLF)	30.0 [20.0; 30.0]	30.0 [20.0; 30.0]	.76
ROM (RR)	45.0 [45.0; 45.0]	45.0 [45.0; 45.0]	.66
ROM (LR)	45.0 [45.0; 45.0]	45.0 [45.0; 45.0]	.11
ODI	42.5 (13.3)	44.5 (12.7)	.45
PCS	39.0 (7.1)	37.5 (7.4)	.3
MCS	40.9 (11.7)	42.3 (12.3)	.58
PCL-5-K	27.3 (14.8)	27.3 (13.6)	.99

The values represent mean (SD)/median [Q1; Q3] or n (%).

BMI = body mass index, EXT = extension, FLX = flexion, LBP = low back pain, LLF = left lateral flexion, LR = left rotation, MCS = mental component summary, NRS = numerical rating scale, ODI = Oswestry disability index, PCL-5-K = posttraumatic stress disorder checklist for DSM-5, PCS = physical component summary, PE = physical examination, Q = quartile, RLF = right lateral flexion, ROM = range of motion, RP = radiating pain, RR = right rotation, SD = standard deviation, T-MSAT = motion-style acupuncture-treatment using traction, VAS = visual analogue scale, WAD = whiplash-associated disorder.

### 3.2. ITT analysis

Table 3 presents the results of the ITT analysis on the outcome values of both groups at different time points and the differences in the primary and secondary outcomes between the 2 groups. Both groups showed a significant decrease in the NRS score: from 6.06 at baseline to 3.89 on day 4-2 (95% confidence interval [CI] 3.47–4.31) in the T-MSAT group and from 5.98 at baseline to 4.72 (95% CI 4.34–5.10) on day 4-2 in the control group. The difference between the T-MSAT and control groups was 0.94 (95% CI 0.40–1.48) on day 4-2, with the T-MSAT group showing a significantly lower NRS score (*P* < .001). The difference between the 2 groups was greater on day 4-2 than at any other measurement point. At discharge and the 12-week follow-up, no statistically significant differences were observed between the 2 groups. Furthermore, compared with the control group, the T-MAST group showed a significant improvement in VAS scores for LBP from the start of the intervention to the primary endpoint (day 4-2). Although this significant difference persisted up to day 4-2, it was no longer evident at discharge. The biggest difference between the 2 groups was found on day 4-2. The T-MSAT group showed significantly greater extension ROM on day 4-2 and discharge, but the difference was only 1.58. The results of the lumbar aROM, excluding flexion and extension, are presented in Supplemental Digital Content (see Table S2, Supplemental Digital Content, http://links.lww.com/MD/M953, which shows the lumbar aROM results).

**Table 3:** Primary and secondary outcomes according to treatment and time since randomization (intention-to-treat analysis).

		Baseline (day 2 before Tx)	Day 3	Day 4-2 (day 4 after Tx)	Discharge	12 weeks
NRS LBP	T-MSAT	6.06 (5.77, 6.35)	4.71 (4.35, 5.06)	3.89 (3.47, 4.31)	3.43 (3.03, 3.82)	2.53 (2.05, 3.02)
	Control	5.98 (5.68, 6.28)	5.27 (4.95, 5.59)	4.72 (4.34, 5.10)	3.69 (3.23, 4.14)	2.43 (1.98, 2.87)
	Difference*		0.60 (0.16, 1.04)	0.94 (0.40, 1.48)	0.40 (−0.15, 0.96)	−0.13 (−0.81, 0.55)
	*P* value	–	.008	<.001	.153	.712
VAS LBP	T-MSAT	61.27 (58.41, 64.13)	45.86 (42.19, 49.53)	37.00 (32.62, 41.38)	31.57 (27.25, 35.89)	–
	Control	60.39 (57.38, 63.39)	53.03 (49.51, 56.55)	45.71 (41.64, 49.78)	35.71 (30.40, 41.02)	–
	Difference*	–	7.41 (2.55, 12.28)	9.71 (3.89, 15.54)	5.92 (−0.40, 12.24)	–
	*P* value	–	.003	.001	.066	–
NRS RP	T-MSAT	4.39 (3.71, 5.06)	3.48 (2.88, 4.07)	2.81 (2.24, 3.37)	2.39 (1.85, 2.92)	1.74 (1.15, 2.34)
	Control	4.76 (4.18, 5.33)	3.83 (3.28, 4.37)	3.21 (2.70, 3.72)	2.22 (1.71, 2.73)	1.65 (1.23, 2.07)
	Difference*	–	0.09 (−0.40, 0.59)	0.11 (−0.47, 0.68)	−0.36 (−0.95, 0.24)	−0.19 (−0.92, 0.53)
	*P* value	–	.706	.717	.234	.597
VAS RP	T-MSAT	43.08 (36.08, 50.08)	32.30 (26.44, 38.17)	25.94 (20.48, 31.40)	22.69 (17.33, 28.05)	–
	Control	47.45 (41.59, 53.31)	38.84 (33.20, 44.48)	30.90 (25.53, 36.26)	20.77 (15.58, 25.95)	–
	Difference*	–	3.13 (−2.17, 8.44)	1.56 (−4.44, 7.57)	−4.34 (−10.46, 1.78)	–
	*P* value	–	.243	.606	.162	–
ODI	T-MSAT	42.46 (38.75, 46.18)	–	–	29.50 (26.33, 32.67)	21.15 (12.74, 29.57)
	Control	44.46 (40.91, 48.00)	–	–	33.30 (29.50, 37.09)	20.68 (17.38, 23.99)
	Difference*	–	–	–	3.10 (−1.19, 7.39)	−2.30 (−11.15, 6.56)
	*P* value	–	–	–	.155	.598
SF-36 (PCS)	T-MSAT	38.99 (37.00, 40.99)	–	–	40.72 (38.85, 42.58)	45.66 (42.64, 48.67)
	Control	37.45 (35.39, 39.52)	–	–	38.12 (36.00, 40.23)	44.61 (42.41, 46.82)
	Difference*	–	–	–	−1.87 (−4.66, 0.92)	0.11 (−3.61, 3.84)
	*P* value	–	–	–	.187	.951
SF-36 (MCS)	T-MSAT	40.95 (37.66, 44.23)	–	–	43.92 (41.09, 46.75)	49.52 (46.09, 52.94)
	Control	42.29 (38.85, 45.73)	–	–	43.97 (40.49, 47.44)	49.38 (46.34, 52.42)
	Difference*	–	–	–	−0.40 (−4.64, 3.84)	−0.20 (−4.89, 4.48)
	*P* value	–	–	–	.851	.931
ROM (FLX)	T-MSAT	65.92 (59.18, 72.65)	80.94 (76.64, 85.24)	82.23 (78.42, 86.05)	87.21 (84.42, 90.01)	–
	Control	65.20 (58.91, 71.50)	77.34 (72.12, 82.55)	78.83 (74.02, 83.63)	83.09 (78.75, 87.43)	–
	Difference*	–	−3.46 (−9.24, 2.31)	−3.85 (−9.17, 1.47)	−4.49 (−9.61, 0.62)	–
	*P* value	–	.237	.154	.085	–
ROM (EXT)	T-MSAT	15.41 (13.41, 17.41)	18.34 (17.25, 19.44)	19.13 (18.45, 19.82)	19.69 (19.25, 20.14)	–
	Control	13.98 (12.34, 15.62)	17.26 (15.91, 18.60)	17.23 (15.91, 18.55)	17.98 (16.84, 19.12)	–
	Difference*	–	−0.71 (−2.36, 0.93)	−1.58 (−3.06, −0.10)	−1.58 (−2.80, −0.36)	–
	*P* value	–	.391	.036	.012	–
PCL-5-K	T-MSAT	27.31 (23.17, 31.45)	–	–	21.83 (17.55, 26.11)	15.92 (11.06, 20.77)
	Control	27.29 (23.47, 31.11)	–	–	20.38 (17.00, 23.76)	15.13 (11.23, 19.03)
	Difference*	–	–	–	−1.48 (−6.17, 3.21)	−0.68 (−6.52, 5.16)
	*P* value	–	–	–	.533	.818
PGIC	T-MSAT	–	–	–	2.37 (2.18, 2.55)	2.31 (2.03, 2.59)
	Control	–	–	–	2.47 (2.21, 2.74)	2.12 (1.87, 2.38)
	Difference*	–	–	–	−0.12 (−0.46, 0.22)	0.17 (−0.22, 0.57)
	*P* value	–	–	–	.492	.382

EXT = extension, FLX = flexion, LBP = low back pain, MCS = mental component summary, NRS = numerical rating scale, ODI = Oswestry disability index, PCL-5-K = posttraumatic stress disorder checklist for DSM-5, PCS = physical component summary, PGIC = patient global impression of change, ROM = range of motion, RP = radiating pain, SF-36 = 36-item short-form survey, T-MSAT = motion-style acupuncture treatment using traction, Tx = treatment, VAS = visual analogue scale.

* Differences are shown as mean (95% confidential interval). Analysis of covariance was conducted to calculate differences and *P* values, with sex and age as covariates.

No significant differences were observed between the 2 groups in all outcomes (NRS score for LBP, VAS score for LBP, ODI, physical component summary [PCS], mental component summary [MCS], PCL-5-K, and PGIC scores) measured at the 12-week follow-up (Table 3).

**Figure 2.**
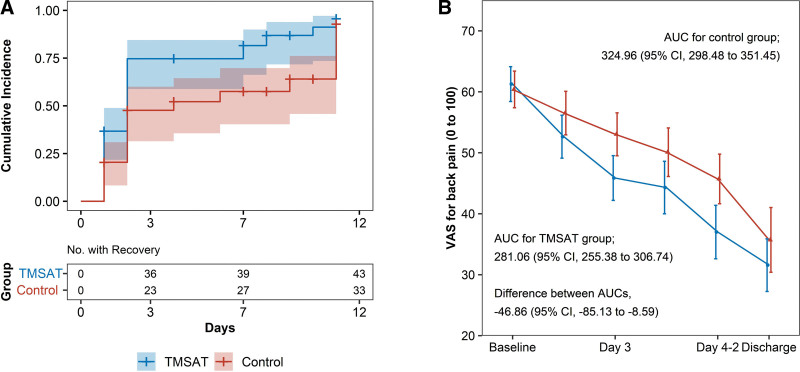
(A) Cox proportional hazard model for the probability of recovery in the T-MSAT and control groups. (B) Changes in the VAS score of back pain outcomes over time and areas under the curves. AUC = area under the curve, CI = confidence interval, T-MSAT = motion-style acupuncture treatment using traction, VAS = visual analogue scale.

Survival analysis of the NRS score for LBP showed that the hazard ratio for achieving a pain reduction < 30% was 1.89 (95% CI: 1.19–3.01) in the T-MSAT group relative to the control group. The logarithmic rank test *P* value was .005, indicating that the T-MSAT group achieved significantly faster pain reduction (Fig. 2A).

When the ITT analysis set was analyzed using the linear mixed model, the T-MSAT group had significantly lower NRS and VAS scores for LBP on days 3, 4-1, and 4-2 compared with the control group (*P* < .05). However, the results for lumbar extension were not significant at all measurement points, unlike other results of the analysis (see Table S3, Supplemental Digital Content, http://links.lww.com/MD/M954, which shows the lumbar aROM results). Overall difference analysis yielded *P* values of .01 for the NRS score for LBP and .013 for the VAS score for LBP, indicating that the NRS and VAS scores decreased significantly faster in the T-MSAT group than in the control group.

### 3.3. AUC analysis

The AUC analysis also showed differences in the rates of LBP and functional recovery between the 2 groups. A significant difference (−46.86 [95% CI, −85.13 to −8.59]) was observed in VAS scores for LBP from baseline to discharge (Fig. 2B). From baseline to the 12-week follow-up, the difference in the AUCs of NRS scores for LBP between the T-MSAT and control groups was −12.59 (95% CI, −57.86 to 32.67), while the difference in ODI scores was −116.70 (95% CI, −601.75 to 368.36). Thus, the AUC of the T-MSAT group continued to be smaller than that of the control group, but the difference was not significant. Furthermore, the difference in PCS scores was 121.96 (95% CI, −111.99 to 355.92), showing that the score increased at a higher rate in the T-MSAT group than in the control group, but the difference was not significant. The difference in extension ROM AUCs was 11.13 (95% CI 2.00–20.25), being significantly higher in the T-MSAT group. AUC analysis results for all outcomes, except VAS score, are shown in Supplemental Digital Content (see Table S4, Supplemental Digital Content, http://links.lww.com/MD/M955, which shows the AUC analysis results).

### 3.4. PP and LOCF analyses

Table 4 presents the results of the PP analysis of the outcome values of both groups at different time points and differences in primary and secondary outcomes between the 2 groups. The differences in NRS scores for LBP between the T-MSAT and control groups on days 3 and 4-2 were 0.67 (95% CI 0.22–1.11) and 0.95 (95% CI 0.43–1.48), respectively, showing a significantly lower NRS score in the T-MSAT group than in the control group. The T-MSAT group also showed a significantly lower VAS score for LBP on days 3 and 4-2. However, this difference diminished, becoming insignificant at discharge and the 12-week follow-up. PP analysis results were generally similar to the ITT analysis results. However, improvement in lumbar extension was significant on day 4-2 and discharge in the ITT analysis, but only at discharge in the PP analysis. The results of the lumbar aROM, excluding flexion and extension, are presented in Supplemental Digital Content (see Table S5, Supplemental Digital Content, http://links.lww.com/MD/M956, which shows the lumbar aROM results). The LOCF analysis results (see Table S6, Supplemental Digital Content, http://links.lww.com/MD/M957, which shows the LOCF analysis results) also showed similar patterns to those observed in the ITT and PP analysis results.

**Table 4:** Primary and secondary outcomes according to treatment and time since randomization (per-protocol analysis).

		Baseline (day 2 before Tx)	Day 3	Day 4-2 (day 4 after Tx)	Discharge	12 weeks
NRS LBP	T-MSAT	6.06 (5.77, 6.35)	4.67 (4.29, 5.06)	3.88 (3.47, 4.30)	3.47 (3.04, 3.89)	2.32 (1.86, 2.77)
	Control	5.98 (5.68, 6.28)	5.27 (4.95, 5.59)	4.71 (4.33, 5.09)	3.69 (3.22, 4.15)	2.42 (1.98, 2.86)
	Difference*	–	0.67 (0.22, 1.11)	0.95 (0.43, 1.48)	0.40 (−0.18, 0.97)	0.09 (−0.57, 0.76)
	*P* value	–	.004	<.001	.174	.779
VAS LBP	T-MSAT	61.27 (58.41, 64.13)	45.30 (41.27, 49.33)	37.37 (32.88, 41.87)	31.90 (27.11, 36.68)	–
	Control	60.39 (57.38, 63.39)	53.19 (49.67, 56.71)	45.67 (41.54, 49.79)	35.79 (30.41, 41.18)	–
	Difference*	–	8.11 (3.00, 13.22)	9.23 (3.41, 15.06)	5.66 (−0.99, 12.30)	–
	*P* value	–	.002	.002	.094	–
NRS RP	T-MSAT	4.39 (3.71, 5.06)	3.49 (2.84, 4.14)	2.95 (2.36, 3.54)	2.40 (1.81, 2.98)	1.43 (0.94, 1.92)
	Control	4.76 (4.18, 5.33)	3.83 (3.28, 4.38)	3.21 (2.69, 3.72)	2.23 (1.72, 2.74)	1.64 (1.22, 2.06)
	Difference*	–	0.09 (−0.42, 0.60)	−0.03 (−0.60, 0.54)	−0.35 (−0.97, 0.26)	0.08 (−0.57, 0.73)
	*P* value	–	.732	.914	.253	.808
VAS RP	T-MSAT	43.08 (36.08, 50.08)	32.28 (25.93, 38.63)	27.63 (21.93, 33.32)	22.82 (16.96, 28.68)	–
	Control	47.45 (41.59, 53.31)	39.02 (33.33, 44.71)	30.92 (25.47, 36.36)	20.97 (15.72, 26.21)	–
	Difference*	–	3.15 (−2.40, 8.71)	−0.15 (−6.12, 5.82)	−4.33 (−10.73, 2.06)	–
	*P* value	–	.262	.96	.182	–
ODI	T-MSAT	42.46 (38.75, 46.18)	–	–	28.92 (25.69, 32.15)	19.13 (10.93, 27.32)
	Control	44.46 (40.91, 48.00)	–	–	33.34 (29.53, 37.15)	20.82 (17.47, 24.17)
	Difference*	–	–	–	3.66 (−0.49, 7.82)	−0.27 (−9.00, 8.46)
	*P* value	–	–	–	.083	.95
SF-36 (PCS)	T-MSAT	38.99 (37.00, 40.99)	–	–	43.81 (40.77, 46.84)	51.31 (48.18, 54.43)
	Control	37.45 (35.39, 39.52)	–	–	44.15 (40.66, 47.63)	49.53 (46.55, 52.50)
	Difference*	–	–	–	−0.38 (−4.75, 3.99)	−1.97 (−6.41, 2.47)
	*P* value	–	–	–	.863	.377
SF-36 (MCS)	T-MSAT	40.95 (37.66, 44.23)	–	–	43.81 (40.77, 46.84)	51.31 (48.18, 54.43)
	Control	42.29 (38.85, 45.73)	–	–	44.15 (40.66, 47.63)	49.53 (46.55, 52.50)
	Difference*	–	–	–	−0.38 (−4.75, 3.99)	−1.97 (−6.41, 2.47)
	*P* value	–	–	–	.863	.377
ROM (FLX)	T-MSAT	65.92 (59.18, 72.65)	80.70 (76.02, 85.37)	81.40 (77.15, 85.64)	86.86 (83.70, 90.02)	–
	Control	65.20 (58.91, 71.50)	77.40 (72.16, 82.63)	78.96 (74.24, 83.68)	83.18 (78.90, 87.45)	–
	Difference*	–	−2.90 (−8.85, 3.05)	−2.68 (−8.05, 2.70)	−3.97 (−9.27, 1.33)	–
	*P* value	–	.335	.325	.14	–
ROM (EXT)	T-MSAT	15.41 (13.41, 17.41)	18.37 (17.21, 19.54)	19.07 (18.32, 19.82)	19.65 (19.15, 20.16)	–
	Control	13.98 (12.34, 15.62)	17.29 (15.96, 18.63)	17.29 (15.99, 18.60)	18.04 (16.92, 19.16)	–
	Difference*	–	−0.57 (−2.25, 1.11)	−1.33 (−2.86, 0.19)	−1.38 (−2.64, −0.12)	–
	*P* value	–	.503	.086	.032	–
PCL-5-K	T-MSAT	27.31 (23.17, 31.45)	–	–	20.87 (16.42, 25.31)	12.97 (8.75, 17.19)
	Control	27.29 (23.47, 31.11)	–	–	20.25 (16.84, 23.66)	14.79 (10.95, 18.62)
	Difference*	–	–	–	−0.46 (−4.95, 4.03)	1.86 (−3.19, 6.92)
	*P* value	–	–	–	.839	.464
PGIC	T-MSAT	–	–	–	2.36 (2.18, 2.54)	2.21 (1.97, 2.45)
	Control	–	–	–	2.47 (2.20, 2.74)	2.12 (1.85, 2.38)
	Difference*	–	–	–	−0.12 (−0.47, 0.22)	0.10 (−0.27, 0.47)
	*P* value	–	–	–	.481	.605

EXT = extension, FLX = flexion, LBP = low back pain, MCS = mental component summary, NRS = numerical rating scale, ODI = Oswestry disability index, PCL-5-K = posttraumatic stress disorder checklist for DSM-5, PCS = physical component summary, PGIC = patient global impression of change, ROM = range of motion, RP = radiating pain, SF-36 = 36-item short-form survey, T-MSAT = motion-style acupuncture treatment using traction, Tx = treatment, VAS = visual analogue scale.

* Differences are shown as mean (95% confidential interval). Analysis of covariance was conducted to calculate differences and *P* values, with sex and age as covariates.

### 3.5. Subgroup analysis

Subgroup analysis was conducted according to sex, age, body mass index, whiplash-associated disorders (WAD), previous LBP history, ODI score, time of onset, ROM, and PCL-5-K score of the T-MSAT and control groups. Analysis according to WAD showed a significant difference (*P* = .03), while no significant differences were observed according to other factors.

### 3.6. Comparison of primary and secondary outcomes between the 2 groups according to admission date

The mean and median lengths of hospital stay were, respectively, 7.8 (2.8) and 8 (6–10) days in the T-MSAT group and 8.3 (2.7) and 8.5 (6.75–11) days in the control group. The percentages of patients discharged up to the eighth day were 60.4% and 50.0% in the T-MSAT and control groups, respectively, showing a difference of 10.4%. The percentages of patients discharged up to the ninth day were 70.8% and 58.3% in the T-MSAT and control groups, respectively, showing a difference of 12.5%. These results indicate a slightly earlier discharge tendency in the T-MSAT group.

No significant differences were observed in NRS scores for LBP according to the length of hospital stay. A significant difference (9.58 [95% CI 0.10–19.06]) was observed in the VAS score for LBP between the T-MSAT and control groups among patients discharged on day 7. Lumbar flexion was −10.54 (95% CI −20.90 to −0.19) on the sixth day. Lumbar extension showed a significant difference from the sixth (*P* = .019) to the twelfth day (*P* = .012). Meanwhile, the ODI showed a significant difference (6.45 [95% CI 0.55–12.35]) on the eighth day. However, the PCS showed a significant difference (−2.96 [−5.72 to −0.19; *P* = .036]) only on the tenth day (see Table S7, Supplemental Digital Content, http://links.lww.com/MD/M958, which shows the difference in these scores).

No significant differences were observed in the accident settlement status, time to settlement, treatment completion status, time to treatment completion, and return to work status between the 2 groups.

### 3.7. Co-interventions

During the study period, 38.8% and 28.2% of the participants in the T-MSAT and control groups, respectively, used analgesics. The medications used were primarily analgesics, muscle relaxants, and herbal granules.

### 3.8. AEs

AEs were reported by 36 patients: 18 each in the T-MSAT and control groups. However, these AEs were not related to the clinical trial, and all were mild, requiring no specific treatment. The reported AEs included headache (n = 28), nausea (n = 5), cold (n = 2), indigestion (n = 4), exacerbation of LBP (n = 1), dizziness (n = 2), fever (n = 2), LBP (n = 2), neck pain (n = 1), pantalgia (n = 2), constipation (n = 1), and urticaria (n = 1). Among these, headache and pantalgia persisted in each participant with these AEs after trial completion, while all other AEs persisted for 2 (1–5) days. Most of the AEs involved mild symptoms, such as headache, indigestion, and body ache, commonly associated with TAs.

## 4. Discussion

Several RCTs have investigated the application of MSAT, including one that reported improvements in cervical pain and dysfunction in patients with whiplash injury after a TA.^[17]^ Another multicentre RCT demonstrated that MSAT in patients with acute LBP and severe dysfunction produced a therapeutic effect more than 5 times greater than that of conventional analgesic injection.^[16]^ Additional RCTs showed greater pain reduction and ROM improvement with MSAT than with loxoprofen or physical therapy in patients with acute LBP.^[26,27]^ The findings of the aforementioned studies and the present study confirm that MSAT significantly improves initial pain in patients with acute musculoskeletal pain.

The T-MSAT utilized in this study is similar to the MSAT employed by Shin et al However, the present study utilized a specially designed device to apply traction to the body of the patient, rather than relying on manual application by a person. A previous study applied T-MSAT and tested its effects on patients with lumbar dysfunction [29]; however, that study had several limitations, including the retrospective analysis of medical records and unclear classification of LBP. While T-MSAT is mainly used in clinical practice for acute LBP or spinal diseases with severe disability, no clinical studies had been conducted thus far. Consequently, this is the first RCT to assess the effectiveness and safety of T-MSAT, focusing on acute LBP that manifests after a TA.

The present study showed that NRS and VAS scores for LBP were significantly lower in the T-MSAT group than in the control group at the primary endpoint. The difference in NRS scores for LBP between the 2 groups was highest on day 4-2 (0.94 [0.40–1.48]). The results of the comparison between days 4-1 and 4-2, that is, before and after treatment on the same day, showed that the effects of T-MSAT appeared most clearly immediately after treatment. However, no differences were observed in NRS and VAS scores for LBP between the 2 groups at discharge. This could be attributed to the insufficient number of T-MSAT sessions. The present study included only 3 treatment sessions, whereas the mean number of treatment sessions in a previous retrospective chart review study was 7.29.^[28]^ The NRS scores of radiating leg pain did not significantly differ between the 2 groups, possibly because some patients had no radiating leg pain and others had mild pain. The daily difference in ODI and SF-12 scores was thought to be of no clinical significance; however, these scores were only assessed at baseline, discharge, and the 12-week follow-up. Furthermore, the ODI, PCS, and MCS scores did not differ significantly between the groups at discharge and the 12-week follow-up. Moreover, although ROM in the T-MSAT group was slightly greater than that in the control group, the difference was not statistically significant. Future MSAT research, building on our findings, must ensure observation of early changes in function and quality of life.

Unlike previous studies that did not apply criteria for distinguishing between acute and chronic cases,^[28]^ establishing the cause based solely on the time of onset,^[16]^ we recruited participants meeting a clearly defined criterion for acute LBP (occurring within 1 week of a TA). The log-rank test and AUC analysis further confirmed the efficacy of T-MSAT in achieving rapid pain reduction for acute LBP. Furthermore, the length of hospital stay was slightly shorter in the T-MSAT group, indicating that participants in this group were able to resume their daily activities quicker than those in the control group. Investigation of discharged patients from admission to the ninth day showed a 12.5% difference in discharge rates, suggesting that T-MSAT contributed to faster discharge.

The analysis based on WAD revealed the superior efficacy of T-MSAT compared with the control group treatment among patients with grade 2 WAD. A previous study on WAD reported that symptoms tend to persist for 3 weeks in grade 1 WAD and more than 6 weeks in grade 2 or 3 WAD.^[29]^ Furthermore, another study highlighted the significance of initiating treatment within the first 3 weeks to prevent the condition from progressing to a chronic stage. The study also noted that grade 1 WAD could progress to grade 2 or 3 without proper treatment.^[29]^ Therefore, early intervention for symptoms resulting from a TA considerably impacts prognosis. A significant interaction (*P* = .03) was observed in the subgroup analysis based on WAD. The findings of the present study suggest that T-MSAT may be more effective in patients with higher grade WAD than in those with lower grade WAD. Given the ability of T-MSAT to quickly reduce initial pain, it is anticipated to prevent the exacerbation of WAD to higher grades and positively impact prognosis.

This study showed that the disparity in therapeutic effects between the 2 groups, which was evident immediately after T-MSAT treatment, had diminished by the time of discharge. Thus, no significant difference was observed between the 2 groups at the 12-week follow-up. Furthermore, a previous study reported that the TA-related settlement status and treatment completion status are associated with clinical symptoms after a TA.^[30]^ This study lasted 12 weeks, including follow-up, to monitor clinical progress related to LBP, as well as the treatment completion status, accident settlement status, and return to work. However, no differences were observed between the 2 groups. Although T-MSAT contributed to a quicker initial recovery, factors such as return to work or accident settlement status may be closely associated with the socioeconomic characteristics of the patient and the TA insurance system in Korea.

This study had some limitations. First, the design did not permit blinding of the treatment applicator. To address this, the assessor was separated and blinded. Second, the expectancy score for T-MSAT, investigated at the start of the trial, was significantly lower in the experimental group than in the control group. Given the potential influence of expectancy on outcomes owing to the placebo effect, the significantly lower expectancy score in the experimental group might have negatively impacted the findings. Third, the patient response rate in the additional follow-up survey was low. Settlement for TAs is reached within 45 days in most cases, and most patients would assume that all issues associated with the TA would have been resolved after settlement or treatment completion. Consequently, adherence to the 12-week follow-up may have been low. Furthermore, the study was limited by the lack of validated devices, such as a goniometer,^[31,32]^ for measuring ROM. However, measurements were conducted by a Korean medicine doctor who underwent training prior to the trial to minimize potential bias.

The approved protocol for this trial defined recovery as a ≥ 50% pain reduction,^[33]^ but the actual analysis was performed with recovery defined as a ≥ 30% pain reduction. This was based on opinions of clinicians who felt that ≥ 50% pain reduction was an excessively strict criterion considering the general length of hospital stay and the follow-up period.

The T-MSAT group showed a higher co-intervention ratio than the control group (38.8% vs 28.2%), indicating more frequent prescriptions of analgesics and muscle relaxants in the T-MSAT group. Individuals initially prescribed analgesics after a car accident were more often assigned to the T-MSAT group. However, during follow-up, a higher percentage of participants in the T-MSAT group (12.2%) continued utilizing analgesic medications than in the control group (6.1%). This presents a potential limitation and confounding factor for assessing effectiveness.

Furthermore, discharge times varied between the 2 groups, making comparisons under the same conditions challenging. The control group had more patients with longer hospital stays than the T-MSAT group; consequently, participants in the control group had received more treatments than those in the T-MSAT group before discharge. To mitigate such bias, the study compared the results of patients within specific discharge days. However, future studies should establish a standardized time point for comparison, rather than using the discharge time. Additionally, the study population was restricted to inpatients with acute LBP resulting from a TA, limiting the generalizability of the findings to all patients with LBP.

As this study confirmed the potential for fast recovery after treatment in patients with acute LBP, future studies evaluating the therapeutic effects of T-MSAT in patients with nontraumatic LBP or chronic LBP are warranted. Additionally, well-designed studies should investigate the effects of T-MSAT on WAD grade or severity and its long-term effects.

This RCT demonstrated the effectiveness and safety of T-MSAT, which had not been previously studied. The findings demonstrated that T-MSAT can achieve fast pain reduction in patients with acute LBP after a TA. Furthermore, the findings also suggested that T-MSAT may be slightly more effective when applied to patients with severe acute LBP, such as grade 2 WAD, than in those with mild acute LBP. Further, this study showed that combined treatment with IKMT and T-MSAT was effective in reducing initial pain in patients with acute LBP caused by a TA. Nevertheless, well-designed large-scale studies are warranted to explore the use of T-MSAT in patients with severe spinal disease.

## Author contributions

**Conceptualization:** Byung-Hak Park, Jeong-Hun Han.

**Data curation:** Jin-Hun Park, Tae-Woon Min, Hyun-Jun Lee.

**Formal analysis:** Yoon Jae Lee, Sook-Hyun Lee, Kyoung Sun Park.

**Investigation:** Yoon Jae Lee, Sook-Hyun Lee, Kyoung Sun Park.

**Writing – original draft:** Byung-Hak Park, Jeong-Hun Han.

**Writing – review & editing:** Yoon Jae Lee, In-Hyuk Ha.

## Supplementary Material














