# Supplementary material for: Effectiveness and safety of motion-style acupuncture treatment using traction for inpatients with acute low back pain caused by a traffic accident: A randomized controlled trial
Source: Medicine (Baltimore). 2024 Jun 21;103(25):e38590. doi: 10.1097/MD.0000000000038590 (PMC11191944; doi:10.1097/MD.0000000000038590)
Supplement: Supplementary file 1 [file medi-103-e38590-s001.docx]

# Effectiveness and safety of motion-style acupuncture treatment using traction for inpatients with acute low back pain caused by a traffic accident: A randomised controlled trial

# Byung-Hak Park, Jeong-Hun Han, Jin-Hun Park, Tae-Woon Min, Hyun-Jun Lee, Yoon Jae Lee, Sook-Hyun Lee, Kyoung Sun Park, In-Hyuk Ha

**Supplemental Digital Content 1. Acupuncture treatment reported in accordance with STRICTA standards**

| **STRICTA checklist items** |  | **Acupuncture of T-MSAT** |  | **Acupuncture of IKMT** |
| --- | --- | --- | --- | --- |
| Acupuncture rationale | **1a) Style of acupuncture** | Korean style | **1a) Style of acupuncture** | Korean style |
|  | **1b) Reasoning for treatment provided** | Acupoints based on MSAT | **1b) Reasoning for treatment provided** | Acupoints based on Korean Medicine principles |
| Details of needling | **2a) Number of needle insertions per subject per session** | 7 | **2a) Number of needle insertion per subject per session (range)** | 6-12 |
|  | **2b) Names of points used** | LI11(bilateral), LR2(bilateral), EX-B2(bilateral), and GV16(unilateral) | **2b) Names of points used** | BL23(bilateral), BL54(bilateral), SP6(unilateral), GB38(unilateral) and Ashi |
|  | **2c) Depth of insertion**  **(cm)** | 1 | **2c) Depth of insertion**  **(cm)** | 0.6-1.2 |
|  | **2d) Responses sought** | De qi sensation | **2d) Responses sought** | De qi sensation |
|  | **2e) Needle stimulation** | Holding and twisting (捻轉) | **2e) Needle stimulation** | Holding and twisting (捻轉), Lifting and thrusting (提揷), electrical stimulation |
|  | **2f) Needle retention time (minutes)** | 15 | **2f) Needle retention time (minutes)** | 15-20 |
|  | **2g) Needle type** | 0.30×0.40 mm disposable acupuncture (Dongbang Medical, Boryeong, Korea) | **2g) Needle type** | 0.30×0.40 mm disposable acupuncture (Dongbang Medical, Boryeong, Korea) |
| Treatment Regimen | **3a) Number of treatment sessions** | 3 | **3a) Number of treatment sessions** | 14-28 sessions (depending on the hospital stay, two sessions/days) |
|  | **3b) Frequency of treatment sessions (sessions/week)** | once /day | **3b) Frequency of treatment sessions (sessions/week)** | 14 |
|  | **3b) Duration of treatment sessions (minutes)** | 15 | **3b) Duration of treatment sessions (minutes)** | 15-20 |
| Other components of treatment | **4a) Other interventions administered** | TMSAT | **4a) Other interventions administered** | Chuna therapy, pharmacopuncture, herbal medicine |
| Practitioner background | **5) Description of participating acupuncturists** | A doctor of Korean medicine with over 18 months of clinical experience | **5) Description of participating acupuncturists** | A doctor of Korean medicine with over 18 months of clinical experience |

STRICA, Standards for reporting interventions in clinical trials of acupuncture; T-MAST, motion-style acupuncture treatment using traction, IKMT, integrative Korean medicine treatment
