# Supplementary material for: Effectiveness and safety of motion-style acupuncture treatment using traction for inpatients with acute low back pain caused by a traffic accident: A randomized controlled trial
Source: Medicine (Baltimore). 2024 Jun 21;103(25):e38590. doi: 10.1097/MD.0000000000038590 (PMC11191944; doi:10.1097/MD.0000000000038590)
Supplement: Supplementary file 2 [file medi-103-e38590-s002.docx]

# Effectiveness and safety of motion-style acupuncture treatment using traction for inpatients with acute low back pain caused by a traffic accident: A randomised controlled trial

# Byung-Hak Park, Jeong-Hun Han, Jin-Hun Park, Tae-Woon Min, Hyun-Jun Lee, Yoon Jae Lee, Sook-Hyun Lee, Kyoung Sun Park, In-Hyuk Ha

# Supplemental Digital Content 2. Range of motion (intention-to-treat analysis)

|  | **Baseline**  **(day 2 before Tx)** | **Day 2-2**  **(day 2 after Tx)** | **Day 3** | **Day 4-1**  **(day 4 before Tx)** | **Day 4-2**  **(day 4 after Tx)** | **Discharge** | **12 weeks** | |
| --- | --- | --- | --- | --- | --- | --- | --- | --- |
| **ROM (RLF)** |  |  |  |  |  |  | | - |
| T-MSAT | 25.20 (23.10, 27.30) | 28.37 (27.18, 29.56) | 29.10 (28.27, 29.94) | 29.19 (28.31, 30.08) | 29.49 (28.78, 30.20) | 29.80 (29.40, 30.20) | | - |
| Control | 24.49 (22.50, 26.47) | 27.86 (26.49, 29.23) | 28.04 (26.64, 29.44) | 27.55 (25.81, 29.28) | 28.56 (27.24, 29.88) | 29.37 (28.45, 30.29) | | - |
| Difference^*^ | - | -0.15 (-1.69, 1.40) | -0.69 (-2.24, 0.86) | -1.28 (-3.26, 0.70) | -0.81 (-2.38, 0.76) | -0.25 (-1.28, 0.78) | | - |
| *P* value | - | .85 | .376 | .202 | .307 | .625 | | - |
| **ROM (LLF)** |  |  |  |  |  |  | | - |
| T-MSAT | 25.61 (23.62, 27.61) | 28.37 (27.11, 29.63) | 29.06 (28.21, 29.91) | 29.26 (28.42, 30.10) | 29.49 (28.78, 30.20) | 29.59 (29.03, 30.15) | | - |
| Control | 25.31 (23.36, 27.25) | 27.55 (26.01, 29.09) | 27.90 (26.33, 29.46) | 27.54 (25.80, 29.27) | 28.19 (26.66, 29.72) | 29.14 (28.15, 30.13) | | - |
| Difference^*^ | - | -0.60 (-2.45, 1.24) | -0.92 (-2.57, 0.74) | -1.38 (-3.30, 0.55) | -1.14 (-2.90, 0.61) | -0.25 (-1.37, 0.88) | | - |
| *P* value | - | .518 | .273 | .158 | .198 | .663 | | - |
| **ROM (RR)** |  |  |  |  |  |  | | - |
| T-MSAT | 42.14 (40.16, 44.12) | 44.18 (43.18, 45.19) | 44.18 (43.03, 45.34) | 44.18 (43.03, 45.34) | 44.69 (44.09, 45.29) | 45.00 (45.00, 45.00) | | - |
| Control | 42.65 (40.00, 45.30) | 44.08 (43.06, 45.10) | 44.59 (43.96, 45.22) | 44.39 (43.55, 45.23) | 44.69 (44.09, 45.29) | 44.68 (44.05, 45.31) | | - |
| Difference^*^ | - | -0.20 (-1.54, 1.13) | 0.19 (-1.07, 1.45) | 0.09 (-1.40, 1.58) | 0.04 (-0.84, 0.92) | -0.41 (-1.05, 0.24) | | - |
| *P* value | - | .765 | .765 | .902 | .922 | .217 | | - |
| **ROM (LR)** |  |  |  |  |  |  | | - |
| T-MSAT | 43.37 (41.67, 45.07) | 44.49 (43.67, 45.31) | 44.18 (43.03, 45.34) | 44.18 (43.03, 45.34) | 44.69 (44.09, 45.29) | 44.69 (44.09, 45.29) | | - |
| Control | 42.55 (39.87, 45.23) | 43.78 (42.61, 44.94) | 44.55 (43.86, 45.23) | 44.29 (43.43, 45.14) | 44.69 (44.09, 45.29) | 44.68 (44.05, 45.31) | | - |
| Difference^*^ | - | -0.41 (-1.74, 0.93) | 0.49 (-0.87, 1.86) | -0.03 (-1.53, 1.46) | -0.01 (-0.89, 0.87) | 0.06 (-0.77, 0.89) | | - |
| *P* value | - | .544 | .472 | .964 | .985 | .885 | | - |

*Differences are shown as mean (95% confidential interval). Analysis of covariance was conducted to calculate differences and *P* values, with sex and age as covariates.
LLF, left lateral flexion; LR, left rotation; RLF, right lateral flexion; ROM, range of motion; RR, right rotation; T-MSAT, motion-style acupuncture treatment using traction; Tx, treatment
