# Supplementary material for: Effectiveness and safety of motion-style acupuncture treatment using traction for inpatients with acute low back pain caused by a traffic accident: A randomized controlled trial
Source: Medicine (Baltimore). 2024 Jun 21;103(25):e38590. doi: 10.1097/MD.0000000000038590 (PMC11191944; doi:10.1097/MD.0000000000038590)
Supplement: Supplementary file 3 [file medi-103-e38590-s003.docx]

# Effectiveness and safety of motion-style acupuncture treatment using traction for inpatients with acute low back pain caused by a traffic accident: A randomised controlled trial

# Byung-Hak Park, Jeong-Hun Han, Jin-Hun Park, Tae-Woon Min, Hyun-Jun Lee, Yoon Jae Lee, Sook-Hyun Lee, Kyoung Sun Park, In-Hyuk Ha

**Supplemental Digital Content 3. Primary and secondary outcomes according to treatment and time since randomisation (linear mixed model)**

|  | **Baseline**  **(day 2 before Tx)** | **Day 2-2**  **(day 2 after Tx)** | **Day 3** | **Day 4-1**  **(day 4 before Tx)** | **Day 4-2**  **(day 4 after Tx)** | **Discharge** | **12 weeks** | **Overall difference** |
| --- | --- | --- | --- | --- | --- | --- | --- | --- |
| **NRS LBP** |  |  |  |  |  |  |  |  |
| T-MSAT | 6.06 (5.77, 6.35) | 5.39 (5.04, 5.73) | 4.69 (4.33, 5.05) | 4.56 (4.18, 4.94) | 3.89 (3.45, 4.33) | 3.42 (3.02, 3.82) | 2.58 (2.08, 3.07) | 0.010 |
| Control | 5.98 (5.68, 6.28) | 5.61 (5.27, 5.95) | 5.27 (4.95, 5.59) | 5.04 (4.69, 5.40) | 4.71 (4.33, 5.09) | 3.69 (3.22, 4.15) | 2.44 (1.99, 2.88) |  |
| Difference | _ | 0.31 (-0.21, 0.83) | 0.66 (0.13, 1.18) | 0.55 (0.03, 1.08) | 0.88 (0.35, 1.41) | 0.35 (-0.18, 0.87) | 0.02 (-0.54, 0.57) |  |
| *P* value | _ | .253 | .015 | .04 | .001 | .2 | .955 |  |
| **NRS RP** |  |  |  |  |  |  |  |  |
| T-MSAT | 4.39 (3.71, 5.06) | 4.06 (3.40, 4.72) | 3.46 (2.85, 4.06) | 3.21 (2.62, 3.79) | 2.84 (2.26, 3.43) | 2.38 (1.83, 2.92) | 1.70 (1.06, 2.34) | 0.496 |
| Control | 4.76 (4.18, 5.33) | 4.14 (3.56, 4.73) | 3.83 (3.28, 4.38) | 3.60 (3.05, 4.16) | 3.21 (2.69, 3.72) | 2.23 (1.72, 2.74) | 1.62 (1.16, 2.07) |  |
| Difference | _ | -0.29 (-0.93, 0.36) | -0.01 (-0.66, 0.64) | 0.01 (-0.64, 0.66) | -0.10 (-0.75, 0.56) | -0.53 (-1.18, 0.12) | -0.45 (-1.14, 0.24) |  |
| *P* value | _ | .391 | .981 | .97 | .773 | .115 | .202 |  |
| **VAS LBP** |  |  |  |  |  |  |  |  |
| T-MSAT | 61.27 (58.41, 64.13) | 52.63 (49.12, 56.15) | 45.69 (41.97, 49.40) | 44.46 (40.12, 48.80) | 37.33 (32.73, 41.93) | 31.21 (26.87, 35.56) | - | 0.013 |
| Control | 60.39 (57.38, 63.39) | 56.51 (52.94, 60.08) | 53.19 (49.67, 56.71) | 50.17 (46.15, 54.19) | 45.67 (41.54, 49.79) | 35.13 (29.77, 40.49) | - |  |
| Difference | _ | 4.76 (-0.35, 9.86) | 8.09 (2.95, 13.23) | 6.30 (1.16, 11.44) | 8.89 (3.70, 14.08) | 4.49 (-0.70, 9.68) | - |  |
| *P* value | _ | .071 | .002 | .018 | <.001 | .093 | - |  |
| **VAS RP** |  |  |  |  |  |  |  |  |
| T-MSAT | 43.08 (36.08, 50.08) | 39.59 (33.12, 46.06) | 32.19 (26.24, 38.14) | 30.27 (24.45, 36.09) | 26.51 (20.86, 32.16) | 22.38 (16.97, 27.79) | - | 0.180 |
| Control | 47.45 (41.59, 53.31) | 42.41 (36.35, 48.46) | 39.02 (33.33, 44.71) | 34.56 (29.00, 40.13) | 30.92 (25.47, 36.36) | 21.22 (15.91, 26.53) | - |  |
| Difference | _ | -1.55 ( -7.28, 4.18) | 2.24 ( -3.53, 8.00) | -0.31 ( -6.07, 5.46) | -1.01 ( -6.83, 4.82) | -5.66 (-11.49, 0.16) | - |  |
| *P* value | _ | .599 | .452 | .918 | .737 | .059 | - |  |
| **ODI** |  |  |  |  |  |  |  |  |
| T-MSAT | 42.46 (38.75, 46.18) | - | - | - | - | 29.26 (26.09, 32.43) | 20.16 (15.12, 25.21) | 0.554 |
| Control | 44.46 (40.91, 48.00) | - | - | - | - | 33.34 (29.53, 37.15) | 21.46 (17.82, 25.11) |  |
| Difference | _ | - | - | - | - | 2.07 (-3.67, 7.81) | -1.23 (-7.27, 4.85) |  |
| *P* value | _ | - | - | - | - | .483 | .694 |  |
| **SF-36 (PCS)** |  |  |  |  |  |  |  |  |
| T-MSAT | 38.99 (37.00, 40.99) |  |  |  |  | 40.75 (38.89, 42.60) | 45.75 (43.14, 48.35) | 0.787 |
| Control | 37.45 (35.39, 39.52) | - | - | - | - | 38.23 (36.11, 40.35) | 44.62 (42.38, 46.86) |  |
| Difference | _ | - | - | - | - | -0.92 (-4.36, 2.52) | 0.28 (-3.31, 3.87) |  |
| *P* value | _ | - | - | - | - | .603 | .879 |  |
| **ROM (FLX)** |  |  |  |  |  |  |  |  |
| T-MSAT | 65.92 (59.18, 72.65) | 73.67 (68.16, 79.19) | 81.04 (76.70, 85.38) | 79.58 (74.83, 84.34) | 81.78 (77.69, 85.86) | 87.19 (84.34, 90.03) | - | 0.848 |
| Control | 65.20 (58.91, 71.50) | 73.98 (68.63, 79.33) | 77.40 (72.16, 82.63) | 77.40 (72.18, 82.62) | 78.96 (74.24, 83.68) | 83.30 (79.09, 87.50) | - |  |
| Difference | - | 1.02 (-5.20, 7.24) | -2.18 (-8.45, 4.07) | -0.72 (-6.99, 5.53) | -1.54 (-7.86, 4.78) | -2.85 (-9.14, 3.43) | - |  |
| *P* value | - | .75 | .499 | .822 | .637 | .378 | - |  |
| **ROM (EXT)** |  |  |  |  |  |  |  |  |
| T-MSAT | 15.41 (13.41, 17.41) | 16.43 (14.92, 17.94) | 18.44 (17.38, 19.50) | 18.65 (17.74, 19.56) | 19.11 (18.39, 19.83) | 19.69 (19.23, 20.14) | - | 0.597 |
| Control | 13.98 (12.34, 15.62) | 16.33 (14.74, 17.91) | 17.29 (15.96, 18.63) | 16.88 (15.40, 18.35) | 17.29 (15.99, 18.60) | 18.19 (17.11, 19.27) | - |  |
| Difference | _ | 1.33 (-0.53, 3.18) | 0.50 (-1.37, 2.37) | -0.12 (-1.99, 1.74) | -0.21 (-2.09, 1.68) | 0.06 (-1.82, 1.93) | - |  |
| *P* value | _ | .166 | .601 | .899 | .832 | .954 | - |  |
| **ROM (RLF)** |  |  |  |  |  |  |  |  |
| T-MSAT | 25.20 (23.10, 27.30) | 28.37 (27.18, 29.56) | 29.17 (28.38, 29.96) | 29.27 (28.45, 30.10) | 29.44 (28.67, 30.22) | 29.79 (29.38, 30.20) | - | 0.865 |
| Control | 24.49 (22.50, 26.47) | 27.86 (26.49, 29.23) | 28.02 (26.60, 29.44) | 27.60 (25.90, 29.31) | 28.65 (27.38, 29.91) | 29.36 (28.45, 30.28) | - |  |
| Difference | - | 0.20 (-1.82, 2.22) | -0.42 (-2.45, 1.61) | -0.94 (-2.97, 1.09) | -0.08 (-2.13, 1.97) | 0.27 (-1.77, 2.30) | - |  |
| *P* value | - | .844 | .689 | .37 | .942 | .799 | - |  |
| **ROM (LLF)** |  |  |  |  |  |  |  |  |
| T-MSAT | 25.61 (23.62, 27.61) | 28.37 (27.11, 29.63) | 29.17 (28.38, 29.96) | 29.27 (28.45, 30.10) | 29.44 (28.67, 30.22) | 29.58 (29.01, 30.15) | - | 0.788 |
| Control | 25.31 (23.36, 27.25) | 27.55 (26.01, 29.09) | 27.92 (26.35, 29.48) | 27.60 (25.90, 29.31) | 28.33 (26.89, 29.77) | 29.15 (28.16, 30.14) | - |  |
| Difference | - | -0.51 (-2.51, 1.49) | -0.93 (-2.95, 1.08) | -1.35 (-3.36, 0.67) | -0.82 (-2.86, 1.21) | -0.15 (-2.17, 1.87) | - |  |
| *P* value | - | .621 | .369 | .194 | .432 | .886 | - |  |
| **ROM (RR)** |  |  |  |  |  |  |  |  |
| T-MSAT | 42.14 (40.16, 44.12) | 44.18 (43.18, 45.19) | 44.17 (42.99, 45.35) | 44.17 (42.99, 45.35) | 44.67 (44.01, 45.32) | 45.00 (45.00, 45.00) | - | 0.972 |
| Control | 42.65 (40.00, 45.30) | 44.08 (43.06, 45.10) | 44.58 (43.94, 45.22) | 44.38 (43.52, 45.23) | 44.69 (44.08, 45.30) | 44.68 (44.06, 45.30) | - |  |
| Difference | - | -0.61 (-2.80, 1.57) | -0.10 (-2.29, 2.10) | -0.31 (-2.50, 1.89) | -0.46 (-2.67, 1.75) | -0.89 (-3.10, 1.31) | - |  |
| *P* value | - | .586 | .93 | .786 | .686 | .43 | - |  |
| **ROM (LR)** |  |  |  |  |  |  |  |  |
| T-MSAT | 43.37 (41.67, 45.07) | 44.49 (43.67, 45.31) | 44.17 (42.99, 45.35) | 44.17 (42.99, 45.35) | 44.67 (44.01, 45.32) | 44.69 (44.08, 45.30) | - | 0.855 |
| Control | 42.55 (39.87, 45.23) | 43.78 (42.61, 44.94) | 44.58 (43.94, 45.22) | 44.27 (43.40, 45.15) | 44.69 (44.08, 45.30) | 44.68 (44.06, 45.30) | - |  |
| Difference | - | 0.10 (-2.04, 2.25) | 1.24 (-0.92, 3.40) | 0.93 (-1.23, 3.08) | 0.81 (-1.36, 2.99) | 0.76 (-1.40, 2.92) | - |  |
| *P* value | - | .926 | .265 | .404 | .469 | .496 | - |  |
| **SF-36 (MCS)** |  |  |  |  |  |  |  |  |
| T-MSAT | 40.95 (37.66, 44.23) | - | - | - | - | 44.10 (41.22, 46.97) | 49.46 (46.16, 52.76) | 0.811 |
| Control | 42.29 (38.85, 45.73) | - | - | - | - | 43.64 (40.17, 47.11) | 49.62 (46.65, 52.59) |  |
| Difference | _ | - | - | - | - | -1.66 (-6.77, 3.45) | -1.05 (-6.38, 4.29) |  |
| *P* value | _ | - | - | - | - | .529 | .703 |  |
| **PCL-5-K** |  |  |  |  |  |  |  |  |
| T-MSAT | 27.31 (23.17, 31.45) | - | - | - | - | 21.32 (16.99, 25.65) | 16.05 (10.89, 21.21) | 0.885 |
| Control | 27.29 (23.47, 31.11) | - | - | - | - | 20.63 (17.17, 24.09) | 15.28 (11.24, 19.33) |  |
| Difference | _ | - | - | - | - | -1.26 (-6.57, 4.07) | -1.03 (-6.62, 4.56) |  |
| *P* value | _ | - | - | - | - | .645 | .719 |  |
| **PGIC** |  |  |  |  |  |  |  |  |
| T-MSAT | - | - | - | - | - | 2.36 (2.18, 2.54) | 2.28 (1.97, 2.58) | 0.164 |
| Control | - | - | - | - | - | 2.48 (2.21, 2.75) | 2.10 (1.84, 2.37) |  |
| Difference | - | - | - | - | - | -0.13 (-0.60, 0.34) | 0.18 (-0.32, 0.69) |  |
| *P* value | - | - | - | - | - | .898 | .782 |  |

EXT, extension; FLX, flexion; LBP, low back pain; LLF, left lateral flexion; LR, left rotation; MCS, mental component summary; NRS, numerical rating scale; ODI, Oswestry disability index; PCL-5-K, posttraumatic stress disorder checklist for DSM-5; PCS, physical component summary; PGIC, patient global impression of change; RLF, right lateral flexion; ROM, range of motion; RP, radiating pain; RR, right rotation; SF-36, 36-item short form survey; T-MSAT, motion-style acupuncture treatment using traction; Tx, treatment; VAS, visual analogue scale
