# Supplementary material for: Effectiveness and safety of motion-style acupuncture treatment using traction for inpatients with acute low back pain caused by a traffic accident: A randomized controlled trial
Source: Medicine (Baltimore). 2024 Jun 21;103(25):e38590. doi: 10.1097/MD.0000000000038590 (PMC11191944; doi:10.1097/MD.0000000000038590)
Supplement: Supplementary file 4 [file medi-103-e38590-s004.docx]

**Effectiveness and safety of motion-style acupuncture treatment using traction for inpatients with acute low back pain caused by a traffic accident: A randomised controlled trial**

Byung-Hak Park, Jeong-Hun Han, Jin-Hun Park, Tae-Woon Min, Hyun-Jun Lee, Yoon Jae Lee, Sook-Hyun Lee, Kyoung Sun Park, In-Hyuk Ha

**Supplemental Digital Content 4. Area under the curve analysis**

|  | **T-MSAT** | **Control** | **Difference** | ***P* value** |
| --- | --- | --- | --- | --- |
| **NRS LBP** | 279.76 (249.96, 309.57) | 289.80 (257.98, 321.63) | -12.59 (-57.86, 32.67) | .582 |
| **NRS RP** | 194.51 (151.95, 237.07) | 185.23 (151.65, 218.82) | 12.28 (-44.00, 68.57) | .666 |
| **VAS LBP** | 281.06 (255.38, 306.74) | 324.96 (298.48, 351.45) | -46.86 (-85.13, -8.59) | .017 |
| **VAS RP** | 199.59 (162.59, 236.58) | 218.33 (184.00, 252.66) | -11.89 (-63.88, 40.11) | .651 |
| **ROM (FLX)** | 597.64 (573.93, 621.35) | 573.98 (546.03, 601.93) | 27.89 (-9.99, 65.78) | .147 |
| **ROM (EXT)** | 136.75 (132.79, 140.72) | 125.34 (117.52, 133.16) | 11.13 (2.00, 20.25) | .017 |
| **ROM (RLF)** | 212.40 (208.25, 216.54) | 206.69 (199.58, 213.79) | 4.57 (-3.93, 13.07) | .288 |
| **ROM (LLF)** | 211.98 (207.16, 216.81) | 205.11 (197.13, 213.09) | 5.68 (-3.97, 15.33) | .245 |
| **ROM (RR)** | 323.14 (319.48, 326.80) | 322.92 (320.50, 325.35) | 0.29 (-4.28, 4.85) | .901 |
| **ROM (LR)** | 322.83 (317.98, 327.68) | 322.65 (320.19, 325.10) | -0.07 (-5.71, 5.58) | .981 |
| **ODI** | 2248.65 (1846.70, 2650.61) | 2403.07 (2145.75, 2660.39) | -116.70 (-601.75, 368.36) | .631 |
| **PCS** | 3790.12 (3621.45, 3958.80) | 3627.08 (3478.24, 3775.91) | 121.96 (-111.99, 355.92) | .302 |
| **MCS** | 4099.20 (3888.46, 4309.94) | 4095.85 (3866.17, 4325.54) | 0.05 (-321.46, 321.56) | 1 |
| **PCL-5-K** | 1674.19 (1325.22, 2023.15) | 1574.54 (1286.91, 1862.16) | 118.94 (-353.33, 591.21) | .618 |

EXT, extension; FLX, flexion; LBP, low back pain; LLF, left lateral flexion; LR, left rotation; MCS, mental component summary; NRS, numerical rating scale; ODI, Oswestry disability index; PCL-5-K, posttraumatic stress disorder checklist for DSM-5; PCS, physical component summary; RLF, right lateral flexion; ROM, range of motion; RP, radiating pain; RR, right rotation; T-MSAT, motion-style acupuncture treatment using traction; VAS, visual analogue scale
