# Supplementary material for: Effectiveness and safety of motion-style acupuncture treatment using traction for inpatients with acute low back pain caused by a traffic accident: A randomized controlled trial
Source: Medicine (Baltimore). 2024 Jun 21;103(25):e38590. doi: 10.1097/MD.0000000000038590 (PMC11191944; doi:10.1097/MD.0000000000038590)
Supplement: Supplementary file 5 [file medi-103-e38590-s005.docx]

**Effectiveness and safety of motion-style acupuncture treatment using traction for inpatients with acute low back pain caused by a traffic accident: A randomised controlled trial**

Byung-Hak Park, Jeong-Hun Han, Jin-Hun Park, Tae-Woon Min, Hyun-Jun Lee, Yoon Jae Lee, Sook-Hyun Lee, Kyoung Sun Park, In-Hyuk Ha

**Supplemental Digital Content 5. Range of motion (per-protocol analysis)**

|  | **Baseline**  **(day 2 before Tx)** | **Day 2-2**  **(day 2 after Tx)** | **Day 3** | **Day 4-1**  **(day 4 before Tx)** | **Day 4-2**  **(day 4 after Tx)** | **Discharge** | **12 weeks** | |
| --- | --- | --- | --- | --- | --- | --- | --- | --- |
| **ROM (RLF)** |  |  |  |  |  |  | |  |
| T-MSAT | 25.20 (23.10, 27.30) | 28.14 (26.79, 29.49) | 29.07 (28.19, 29.95) | 29.19 (28.27, 30.10) | 29.42 (28.61, 30.23) | 29.77 (29.31, 30.22) | | - |
| Control | 24.49 (22.50, 26.47) | 27.81 (26.42, 29.21) | 28.02 (26.60, 29.44) | 27.60 (25.90, 29.31) | 28.65 (27.38, 29.91) | 29.38 (28.47, 30.28) | | - |
| Difference | - | 0.01 (-1.63, 1.64) | -0.67 (-2.29, 0.94) | -1.17 (-3.17, 0.83) | -0.63 (-2.22, 0.96) | -0.21 (-1.27, 0.85) | | - |
| *P* value | - | .993 | .408 | .248 | .435 | .699 | | - |
| **ROM (LLF)** |  |  |  |  |  |  | |  |
| T-MSAT | 25.61 (23.62, 27.61) | 28.14 (26.72, 29.56) | 29.07 (28.19, 29.95) | 29.19 (28.27, 30.10) | 29.42 (28.61, 30.23) | 29.53 (28.90, 30.17) | | - |
| Control | 25.31 (23.36, 27.25) | 27.50 (25.93, 29.07) | 27.92 (26.35, 29.48) | 27.60 (25.90, 29.31) | 28.33 (26.89, 29.77) | 29.17 (28.18, 30.15) | | - |
| Difference | - | -0.48 (-2.47, 1.50) | -0.92 (-2.62, 0.77) | -1.22 (-3.17, 0.74) | -0.90 (-2.63, 0.83) | -0.16 (-1.33, 1.01) | | - |
| *P* value | - | .628 | .282 | .219 | .306 | .783 | | - |
| **ROM (RR)** |  |  |  |  |  |  | |  |
| T-MSAT | 42.14 (40.16, 44.12) | 44.07 (42.93, 45.21) | 44.07 (42.75, 45.38) | 44.07 (42.75, 45.38) | 44.65 (43.97, 45.33) | 45.00 (45.00, 45.00) | | - |
| Control | 42.65 (40.00, 45.30) | 44.06 (43.02, 45.10) | 44.58 (43.94, 45.22) | 44.38 (43.52, 45.23) | 44.69 (44.08, 45.30) | 44.67 (44.03, 45.31) | | - |
| Difference | - | -0.12 (-1.56, 1.31) | 0.27 (-1.09, 1.62) | 0.17 (-1.44, 1.78) | 0.08 (-0.87, 1.03) | -0.42 (-1.12, 0.28) | | - |
| *P* value | - | .864 | .695 | .831 | .866 | .233 | | - |
| **ROM (LR)** |  |  |  |  |  |  | |  |
| T-MSAT | 43.37 (41.67, 45.07) | 44.42 (43.48, 45.35) | 44.07 (42.75, 45.38) | 44.07 (42.75, 45.38) | 44.65 (43.97, 45.33) | 44.65 (43.97, 45.33) | | - |
| Control | 42.55 (39.87, 45.23) | 43.75 (42.56, 44.94) | 44.58 (43.94, 45.22) | 44.27 (43.40, 45.15) | 44.69 (44.08, 45.30) | 44.67 (44.03, 45.31) | | - |
| Difference | - | -0.39 (-1.83, 1.06) | 0.64 (-0.79, 2.07) | 0.05 (-1.56, 1.66) | 0.03 (-0.92, 0.98) | 0.07 (-0.82, 0.97) | | - |
| *P* value | - | .596 | .375 | .951 | .947 | .871 | | - |

LLF, left lateral flexion; LR, left rotation; RLF, right lateral flexion; ROM, range of motion; RR, right rotation; T-MSAT, motion-style acupuncture treatment using traction; Tx, treatment
