# Supplementary material for: Effectiveness and safety of motion-style acupuncture treatment using traction for inpatients with acute low back pain caused by a traffic accident: A randomized controlled trial
Source: Medicine (Baltimore). 2024 Jun 21;103(25):e38590. doi: 10.1097/MD.0000000000038590 (PMC11191944; doi:10.1097/MD.0000000000038590)
Supplement: Supplementary file 6 [file medi-103-e38590-s006.docx]

**Effectiveness and safety of motion-style acupuncture treatment using traction for inpatients with acute low back pain caused by a traffic accident: A randomised controlled trial**

Byung-Hak Park, Jeong-Hun Han, Jin-Hun Park, Tae-Woon Min, Hyun-Jun Lee, Yoon Jae Lee, Sook-Hyun Lee, Kyoung Sun Park, In-Hyuk Ha

**Supplemental Digital Content 6. Primary and secondary outcomes according to treatment and time since randomisation (last observation carried forward)**

|  | **Baseline**  **(day 2 before Tx)** | **Day 2-2**  **(day 2 after Tx)** | **Day 3** | **Day 4-1**  **(day 4 before Tx)** | **Day 4-2**  **(day 4 after Tx)** | **Discharge** | **12 weeks** | |
| --- | --- | --- | --- | --- | --- | --- | --- | --- |
| **NRS LBP** |  |  |  |  |  |  | |  |
| T-MSAT | 6.06 (5.77, 6.35) | 5.39 (5.04, 5.73) | 4.76 (4.38, 5.13) | 4.63 (4.24, 5.03) | 3.96 (3.52, 4.39) | 3.51 (3.08, 3.94) | | 2.71 (2.25, 3.18) |
| Control | 5.98 (5.68, 6.28) | 5.61 (5.27, 5.95) | 5.31 (4.99, 5.63) | 5.08 (4.73, 5.44) | 4.76 (4.37, 5.14) | 3.76 (3.28, 4.23) | | 2.84 (2.34, 3.34) |
| Difference | _ | 0.30 (-0.05, 0.64) | 0.61 (0.17, 1.04) | 0.47 (-0.04, 0.97) | 0.93 (0.39, 1.47) | 0.42 (-0.13, 0.98) | | 0.21 (-0.45, 0.87) |
| *P* value | _ | .089 | .007 | .068 | <.001 | .135 | | .528 |
| **NRS RP** |  |  |  |  |  |  | |  |
| T-MSAT | 4.39 (3.71, 5.06) | 4.06 (3.40, 4.72) | 3.51 (2.91, 4.11) | 3.27 (2.68, 3.85) | 2.92 (2.36, 3.47) | 2.45 (1.90, 3.00) | | 1.90 (1.29, 2.51) |
| Control | 4.76 (4.18, 5.33) | 4.14 (3.56, 4.73) | 3.86 (3.32, 4.40) | 3.63 (3.08, 4.18) | 3.24 (2.74, 3.75) | 2.29 (1.77, 2.80) | | 1.84 (1.37, 2.30) |
| Difference | _ | -0.23 (-0.74, 0.27) | 0.10 (-0.38, 0.58) | 0.04 (-0.50, 0.59) | 0.05 (-0.50, 0.59) | -0.34 (-0.93, 0.25) | | -0.14 (-0.89, 0.61) |
| *P* value | _ | .365 | .691 | .873 | .867 | .251 | | .716 |
| **VAS LBP** |  |  |  |  |  |  | |  |
| T-MSAT | 61.27 (58.41, 64.13) | 52.63 (49.12, 56.15) | 46.24 (42.45, 50.04) | 45.04 (40.64, 49.44) | 37.94 (33.48, 42.40) | 32.06 (27.53, 36.59) | | - |
| Control | 60.39 (57.38, 63.39) | 56.51 (52.94, 60.08) | 53.27 (49.81, 56.72) | 50.31 (46.36, 54.25) | 45.90 (41.83, 49.96) | 36.08 (30.83, 41.34) | | - |
| Difference | _ | 4.59 (0.69, 8.48) | 7.38 (2.55, 12.22) | 5.33 (-0.18, 10.84) | 9.18 (3.45, 14.91) | 6.02 (-0.25, 12.30) | | - |
| *P* value | _ | .021 | .003 | .058 | .002 | .06 | | - |
| **VAS RP** |  |  |  |  |  |  | |  |
| T-MSAT | 43.08 (36.08, 50.08) | 39.59 (33.12, 46.06) | 32.59 (26.71, 38.47) | 30.71 (24.95, 36.48) | 27.31 (22.00, 32.61) | 23.00 (17.63, 28.37) | | - |
| Control | 47.45 (41.59, 53.31) | 42.41 (36.35, 48.46) | 39.24 (33.65, 44.84) | 34.88 (29.39, 40.36) | 31.31 (25.92, 36.70) | 22.71 (17.35, 28.08) | | - |
| Difference | _ | -0.93 (-6.06, 4.20) | 3.35 (-1.81, 8.51) | 0.34 (-5.07, 5.75) | 0.80 (-4.91, 6.51) | -2.44 (-8.62, 3.73) | | - |
| *P* value | _ | .72 | .201 | .901 | .782 | .434 | | - |
| **ODI** |  |  |  |  |  |  | |  |
| T-MSAT | 42.46 (38.75, 46.18) | - | - | - | - | 29.68 (26.47, 32.90) | | 22.31 (17.79, 26.83) |
| Control | 44.46 (40.91, 48.00) | - | - | - | - | 33.72 (29.92, 37.53) | | 23.58 (20.09, 27.07) |
| Difference | _ | - | - | - | - | 3.44 (-0.83, 7.71) | | -0.20 (-5.96, 5.57) |
| *P* value | _ | - | - | - | - | .113 | | .946 |
| **SF-36 (PCS)** |  |  |  |  |  |  | |  |
| T-MSAT | 38.99 (37.00, 40.99) | - | - | - | - | 40.71 (38.93, 42.50) | | 44.43 (42.11, 46.75) |
| Control | 37.45 (35.39, 39.52) | - | - | - | - | 38.26 (36.27, 40.26) | | 42.98 (40.76, 45.20) |
| Difference | _ | - | - | - | - | -1.70 (-4.25, 0.86) | | -0.43 (-3.66, 2.81) |
| *P* value | _ | - | - | - | - | .191 | | .794 |
| **ROM (FLX)** |  |  |  |  |  |  | |  |
| T-MSAT | 65.92 (59.18, 72.65) | 73.67 (68.16, 79.19) | 79.80 (74.90, 84.70) | 78.37 (73.13, 83.60) | 81.02 (76.48, 85.56) | 85.82 (81.95, 89.69) | | - |
| Control | 65.20 (58.91, 71.50) | 73.98 (68.63, 79.33) | 77.65 (72.50, 82.81) | 77.65 (72.52, 82.79) | 79.18 (74.54, 83.83) | 82.96 (78.77, 87.15) | | - |
| Difference | - | 0.43 (-4.75, 5.62) | -1.91 (-7.80, 3.98) | -0.52 (-6.47, 5.42) | -2.16 (-7.57, 3.25) | -3.15 (-8.60, 2.30) | | - |
| *P* value | - | .869 | .521 | .861 | .43 | .254 | | - |
| **ROM (EXT)** |  |  |  |  |  |  | |  |
| T-MSAT | 15.41 (13.41, 17.41) | 16.43 (14.92, 17.94) | 18.16 (17.00, 19.33) | 18.37 (17.32, 19.41) | 18.88 (18.01, 19.75) | 19.39 (18.65, 20.12) | | - |
| Control | 13.98 (12.34, 15.62) | 16.33 (14.74, 17.91) | 17.35 (16.03, 18.66) | 16.94 (15.49, 18.39) | 17.35 (16.07, 18.63) | 18.06 (16.96, 19.16) | | - |
| Difference | _ | 0.84 (-0.88, 2.56) | -0.39 (-2.00, 1.22) | -0.87 (-2.54, 0.81) | -1.14 (-2.64, 0.36) | -1.12 (-2.39, 0.16) | | - |
| *P* value | _ | .336 | .632 | .306 | .136 | .086 | | - |
| **ROM (RLF)** |  |  |  |  |  |  | |  |
| T-MSAT | 25.20 (23.10 to 27.30) | 28.37 (27.18, 29.56) | 29.18 (28.41, 29.96) | 29.29 (28.48, 30.09) | 29.49 (28.78, 30.20) | 29.80 (29.40, 30.20) | | - |
| Control | 24.49 (22.50 to 26.47) | 27.86 (26.49, 29.23) | 28.06 (26.67, 29.46) | 27.65 (25.98, 29.32) | 28.67 (27.44, 29.91) | 29.39 (28.50, 30.27) | | - |
| Difference | - | -0.15 (-1.69, 1.40) | -0.74 (-2.25, 0.76) | -1.23 (-3.08, 0.63) | -0.66 (-2.13, 0.81) | -0.23 (-1.21, 0.75) | | - |
| *P* value | - | .85 | .328 | .192 | .372 | .646 | | - |
| **ROM (LLF)** |  |  |  |  |  |  | |  |
| T-MSAT | 25.61 (23.62 to 27.61) | 28.37 (27.11, 29.63) | 29.18 (28.41, 29.96) | 29.29 (28.48, 30.09) | 29.49 (28.78, 30.20) | 29.59 (29.03, 30.15) | | - |
| Control | 25.31 (23.36 to 27.25) | 27.55 (26.01, 29.09) | 27.96 (26.42, 29.50) | 27.65 (25.98, 29.32) | 28.37 (26.96, 29.78) | 29.18 (28.22, 30.15) | | - |
| Difference | - | -0.60 (-2.45, 1.24) | -0.96 (-2.53, 0.62) | -1.25 (-3.06, 0.56) | -0.92 (-2.52, 0.68) | -0.20 (-1.28, 0.89) | | - |
| *P* value | - | .518 | .231 | .173 | .257 | .722 | | - |
| **ROM (RR)** |  |  |  |  |  |  | |  |
| T-MSAT | 42.14 (40.16 to 44.12) | 44.18 (43.18, 45.19) | 44.18 (43.03, 45.34) | 44.18 (43.03, 45.34) | 44.69 (44.09, 45.29) | 45.00 (45.00, 45.00) | | - |
| Control | 42.65 (40.00 to 45.30) | 44.08 (43.06, 45.10) | 44.59 (43.96, 45.22) | 44.39 (43.55, 45.23) | 44.69 (44.09, 45.29) | 44.69 (44.09, 45.29) | | - |
| Difference | - | -0.20 (-1.54, 1.13) | 0.19 (-1.07, 1.45) | 0.09 (-1.40, 1.58) | 0.04 (-0.84, 0.92) | -0.39 (-1.01, 0.23) | | - |
| *P* value | - | .765 | .765 | .902 | .922 | .213 | | - |
| **ROM (LR)** |  |  |  |  |  |  | |  |
| T-MSAT | 43.37 (41.67 to 45.07) | 44.49 (43.67, 45.31) | 44.18 (43.03, 45.34) | 44.18 (43.03, 45.34) | 44.69 (44.09, 45.29) | 44.69 (44.09, 45.29) | | - |
| Control | 42.55 (39.87 to 45.23) | 43.78 (42.61, 44.94) | 44.59 (43.96, 45.22) | 44.29 (43.43, 45.14) | 44.69 (44.09, 45.29) | 44.69 (44.09, 45.29) | | - |
| Difference | - | -0.41 (-1.74, 0.92) | 0.55 (-0.77, 1.87) | -0.03 (-1.53, 1.46) | -0.01 (-0.89, 0.87) | 0.07 (-0.73, 0.88) | | - |
| *P* value | - | .544 | .407 | .964 | .985 | .854 | | - |
| **SF-36 (MCS)** |  |  |  |  |  |  | |  |
| T-MSAT | 40.95 (37.66, 44.23) | - | - | - | - | 43.84 (41.02, 46.65) | | 48.66 (45.68, 51.64) |
| Control | 42.29 (38.85, 45.73) | - | - | - | - | 43.66 (40.10, 47.23) | | 47.70 (44.72, 50.69) |
| Difference | _ | - | - | - | - | -0.80 (-4.97, 3.36) | | -1.39 (-5.60, 2.82) |
| *P* value | _ | - | - | - | - | .702 | | .513 |
| **PCL-5-K** |  |  |  |  |  |  | |  |
| T-MSAT | 27.31 (23.17, 31.45) | - | - | - | - | 22.10 (17.76, 26.44) | | 17.59 (12.82, 22.36) |
| Control | 27.29 (23.47, 31.11) | - | - | - | - | 20.96 (17.51, 24.41) | | 16.45 (12.63, 20.27) |
| Difference | _ | - | - | - | - | -1.02 (-5.66, 3.62) | | -0.69 (-6.01, 4.63) |
| *P* value | _ | - | - | - | - | .665 | | .797 |
| **PGIC** |  |  |  |  |  |  | |  |
| T-MSAT | - | - | - | - | - | 2.36 (2.19, 2.54) | | 2.31 (2.05, 2.56) |
| Control | - | - | - | - | - | 2.48 (2.22, 2.73) | | 2.13 (1.91, 2.36) |
| Difference | - | - | - | - | - | -0.13 (-0.45, 0.19) | | 0.15 (-0.20, 0.51) |
| *P* value | - | - | - | - | - | .43 | | .398 |

EXT, extension; FLX, flexion; LBP, low back pain; LLF, left lateral flexion; LR, left rotation; MCS, mental component summary; NRS, numerical rating scale; ODI, Oswestry disability index; PCL-5-K, posttraumatic stress disorder checklist for DSM-5; PCS, physical component summary; PGIC, patient global impression of change; RLF, right lateral flexion; ROM, range of motion; RP, radiating pain; RR, right rotation; SF-36, 36-item short form survey; T-MSAT, motion-style acupuncture treatment using traction; Tx, treatment; VAS, visual analogue scale
