# Supplementary material for: Effectiveness and safety of motion-style acupuncture treatment using traction for inpatients with acute low back pain caused by a traffic accident: A randomized controlled trial
Source: Medicine (Baltimore). 2024 Jun 21;103(25):e38590. doi: 10.1097/MD.0000000000038590 (PMC11191944; doi:10.1097/MD.0000000000038590)
Supplement: Supplementary file 7 [file medi-103-e38590-s007.docx]

**Effectiveness and safety of motion-style acupuncture treatment using traction for inpatients with acute low back pain caused by a traffic accident: A randomised controlled trial**

Byung-Hak Park, Jeong-Hun Han, Jin-Hun Park, Tae-Woon Min, Hyun-Jun Lee, Yoon Jae Lee, Sook-Hyun Lee, Kyoung Sun Park, In-Hyuk Ha

**Supplemental Digital Content 7. Subgroup analysis according to admission date**

| Day | **NRS LBP** | | **VAS LBP** | | **ROM (FLX)** | | **ROM (EXT)** | | **ODI** | | **PCS** | |
| --- | --- | --- | --- | --- | --- | --- | --- | --- | --- | --- | --- | --- |
|  | Difference | P value | Difference | P value | Difference | P value | Difference | P value | Difference | P value | Difference | P value |
| 3 | 0.20  (-3.06, 3.45) | 0.876 | 3.54  (-27.60, 34.68) | 0.767 | -5.36  (-52.68, 41.95) | 0.642 | -3.84  (-12.04, 4.37) | 0.257 | 1.13  (-46.67, 48.93) | 0.949 | -3.09  (-22.17, 15.98) | 0.648 |
| 4 | 0.48  (-2.20, 3.15) | 0.677 | 7.31  (-19.96, 34.57) | 0.534 | -4.01  (-25.90, 17.88) | 0.6 | -4.24  (-10.04, 1.57) | 0.119 | 2.74  (-22.77, 28.25) | 0.799 | -4.64  (-18.15, 8.87) | 0.41 |
| 5 | 0.64  (-0.98, 2.26) | 0.409 | 10.21  (-6.75, 27.17) | 0.217 | -9.39  (-23.01, 4.24) | 0.159 | -4.03  (-8.14, 0.07) | 0.054 | 7.43  (-6.67, 21.53) | 0.277 | -3.17  (-10.72, 4.37) | 0.372 |
| 6 | 0.98  (-0.28, 2.24) | 0.121 | 13.10  (-0.28, 26.49) | 0.055 | -10.54  (-20.90, -0.19) | 0.046 | -3.73  (-6.76, -0.71) | 0.019 | 5.89  (-4.31, 16.09) | 0.243 | -2.97  (-8.98, 3.05) | 0.306 |
| 7 | 0.66  (-0.24, 1.56) | 0.146 | 9.58  (0.10, 19.06) | 0.048 | -5.14  (-13.19, 2.91) | 0.202 | -2.23  (-4.27, -0.19) | 0.033 | 6.39  (-0.55, 13.33) | 0.07 | -3.09  (-7.41, 1.23) | 0.153 |
| 8 | 0.60  (-0.16, 1.37) | 0.12 | 7.80  (-0.55, 16.14) | 0.066 | -5.23  (-12.23, 1.78) | 0.14 | -2.15  (-4.00, -0.30) | 0.024 | 6.45  (0.55, 12.35) | 0.033 | -3.41  (-6.97, 0.14) | 0.059 |
| 9 | 0.39  (-0.30, 1.07) | 0.259 | 5.35  (-2.06, 12.75) | 0.153 | -3.62  (-10.05, 2.80) | 0.263 | -1.92  (-3.50, -0.34) | 0.018 | 5.30  (-0.08, 10.68) | 0.053 | -3.19  (-6.39, 0.01) | 0.051 |
| 10 | 0.45  (-0.17, 1.07) | 0.155 | 5.51  (-1.36, 12.38) | 0.114 | -2.80  (-8.21, 2.61) | 0.305 | -1.58  (-2.92, -0.24) | 0.022 | 4.63  (-0.01, 9.27) | 0.051 | -2.96  (-5.72, -0.19) | 0.036 |
| 11 | 0.46  (-0.14, 1.05) | 0.129 | 6.25  (-0.45, 12.94) | 0.067 | -5.57  (-11.26, 0.12) | 0.055 | -1.87  (-3.22, -0.51) | 0.008 | 4.14  (-0.39, 8.66) | 0.073 | -2.56  (-5.23, 0.11) | 0.06 |
| 12 | 0.37  (-0.19, 0.94) | 0.189 | 5.47  (-0.88, 11.83) | 0.091 | -4.56  (-9.75, 0.62) | 0.083 | -1.60  (-2.83, -0.36) | 0.012 | 2.96  (-1.38, 7.29) | 0.178 | -1.59  (-4.39, 1.21) | 0.262 |

EXT, extension; FLX, flexion; LBP, low back pain; NRS, numerical rating scale; ODI, Oswestry disability index; PCS, physical component summary; ROM, range of motion; RP, radiating pain; VAS, visual analogue scale
